# Identification and Characterization of Sterol Acyltransferases Responsible for Steryl Ester Biosynthesis in Tomato

**DOI:** 10.3389/fpls.2018.00588

**Published:** 2018-05-08

**Authors:** Juan A. Lara, Alma Burciaga-Monge, Angel Chávez, Marc Revés, Rodolfo Lavilla, Monserrat Arró, Albert Boronat, Teresa Altabella, Albert Ferrer

**Affiliations:** ^1^Plant Metabolism and Metabolic Engineering Program, Centre for Research in Agricultural Genomics (CRAG) (CSIC-IRTA-UAB-UB), Campus Autonomous University of Barcelona, Cerdanyola del Vallès, Spain; ^2^Laboratory of Medicinal Chemistry, Institute of Biomedicine University of Barcelona, Faculty of Pharmacy and Food Sciences, University of Barcelona, Barcelona, Spain; ^3^Department of Biochemistry and Physiology, Faculty of Pharmacy and Food Sciences, University of Barcelona, Barcelona, Spain; ^4^Department of Biochemistry and Molecular Biomedicine, Faculty of Biology, University of Barcelona, Barcelona, Spain; ^5^Department of Biology, Healthcare, and the Environment, Faculty of Pharmacy and Food Sciences, University of Barcelona, Barcelona, Spain

**Keywords:** Arabidopsis, conjugated sterols, *Solanum lycopersicum*, sterol esterification, stress response, subcellular localization

## Abstract

Steryl esters (SEs) serve as a storage pool of sterols that helps to maintain proper levels of free sterols (FSs) in cell membranes throughout plant growth and development, and participates in the recycling of FSs and fatty acids released from cell membranes in aging tissues. SEs are synthesized by sterol acyltransferases, a family of enzymes that catalyze the transfer of fatty acil groups to the hydroxyl group at C-3 position of the sterol backbone. Sterol acyltransferases are categorized into acyl-CoA:sterol acyltransferases (ASAT) and phospholipid:sterol acyltransferases (PSAT) depending on whether the fatty acyl donor substrate is a long-chain acyl-CoA or a phospolipid. Until now, only Arabidopsis ASAT and PSAT enzymes (AtASAT1 and AtPSAT1) have been cloned and characterized in plants. Here we report the identification, cloning, and functional characterization of the tomato (*Solanum lycopersicum* cv. Micro-Tom) orthologs. SlPSAT1 and SlASAT1 were able to restore SE to wild type levels in the Arabidopsis *psat1-2* and *asat1-1* knock-out mutants, respectively. Expression of SlPSAT1 in the *psat1-2* background also prevented the toxicity caused by an external supply of mevalonate and the early senescence phenotype observed in detached leaves of this mutant, whereas expression of SlASAT1 in the *asat1-1* mutant revealed a clear substrate preference of the tomato enzyme for the sterol precursors cycloartenol and 24-methylene cycloartanol. Subcellular localization studies using fluorescently tagged SlPSAT1 and SlASAT1 proteins revealed that SlPSAT1 localize in cytoplasmic lipid droplets (LDs) while, in contrast to the endoplasmic reticulum (ER) localization of AtASAT1, SlASAT1 resides in the plasma membrane (PM). The possibility that PM-localized SlASAT1 may act catalytically *in trans* on their sterol substrates, which are presumably embedded in the ER membrane, is discussed. The widespread expression of *SlPSAT1* and *SlASAT1* genes in different tomato organs together with their moderate transcriptional response to several stresses suggests a dual role of SlPSAT1 and SlASAT1 in tomato plant and fruit development and the adaptive responses to stress. Overall, this study contributes to enlarge the current knowledge on plant sterol acyltransferases and set the basis for further studies aimed at understanding the role of SE metabolism in tomato plant growth and development.

## Introduction

Sterols are essential eukaryotic cell components that occur in free form (FSs) and conjugated as steryl esters (SEs), steryl glycosides (SGs), and acylated steryl glycosides (ASGs). In SGs, the hydroxyl group at the C3 position of the sterol backbone is linked through a glycosidic bond to a sugar moiety, usually a single glucose residue, whereas in ASGs, the sugar residue at the C3 position carries a fatty acid esterified to the hydroxyl group at C-6 position. By contrast, in SEs, the hydroxyl group of the sterol molecule is directly esterified to a fatty acid (Ferrer et al., 2017). Conjugated sterols usually have a sterol composition that mimics that of major sterols found in the FS fraction. Thereby, in plants, the main sterol moieties found in the SE fraction are β-sitosterol, stigmasterol, and campesterol, and in some species, also cholesterol. However, other less abundant sterols or sterol biosynthetic intermediates are also found in esterified form, and in some cases, esters of sterol precursors may even predominate in the SE fraction. The fatty acid moiety in SEs also shows a wide diversity of species covering a wide range of lengths from C12 to C22, being palmitic, stearic, oleic, linoleic, and linolenic acids the most common ones (Ferrer et al., 2017, and the references cited therein).

It is widely accepted that SEs serve as a storage pool of sterols that helps to maintain proper levels of FSs in cell membranes during plant growth and development. This is achieved through a delicate balance between SE formation and hydrolysis that prevents the potential destabilizing effect that changes of FS levels above or below a certain threshold might have on membrane structure and function (Schaller, 2004; Bouvier-Navé et al., 2010). As key structural components of cell membranes, changes in FS levels and composition have a profound impact on membrane biophysical properties such as permeability, fluidity, and rigidity, and hence largely determine membrane function and the activity of membrane-bound proteins (Hartmann, 1998; Roche et al., 2008; Grosjean et al., 2015). The buffering effect of sterol esterification on FS levels becomes particularly evident when the metabolic flux through the sterol biosynthetic pathway is enhanced, since FS levels remain virtually unchanged while sterols produced in excess accumulate mainly as SEs in cytoplasmic lipid droplets (LDs; Maillot-Vernier et al., 1991; Gondet et al., 1994; Wilkinson et al., 1994; Schaller et al., 1995; Bouvier-Navé et al., 2010). The SE pool is also thought to be a reservoir of FSs that can be mobilized to provide the FSs needed to meet the demand for active plasma membrane (PM) synthesis during rapid tissue and cell growth. Thus, the accumulation of SEs reported to occur during seed maturation (Dyas and Goad, 1993; Harker et al., 2003) would be necessary to supply the FSs required for seedling growth during the early stages of development, and the high levels of SEs found in pollen grains (Hernández-Pinzón et al., 1999) would be important to provide enough FSs to sustain germination and the very fast pollen tube growth once pollen grains land on the female reproductive organ. SEs are also enriched in senescing tissues (Duperon et al., 1984; Bouvier-Navé et al., 2010; Li et al., 2016) where they appear to participate in the recycling of FSs and the fatty acids of phospholipids released from senescing cell membranes. In aging tissues, sterols and fatty acids released from disorganized membranes would be converted to SEs for subsequent transport to other tissues (Holmer et al., 1973; Chen et al., 2007). Relatively high levels of SEs have also been reported in the phloem sap, being esterified cholesterol the largely dominant species (Behmer et al., 2013), but the biological significance of this finding remains to be established. The reason behind a drastic 12-fold increase of SE content observed during ripening of tomato fruits is also unclear, although this response seems to be primarily associated with ripening rather than with aging because the levels of SEs remain virtually unchanged in the non-ripening tomato mutants *rin* and *nor* (Whitaker, 1988). Recently, it has been reported that the lack of SEs in the Arabidopsis *erp1* mutant correlates with an altered response against invasive filamentous pathogens (Kopischke et al., 2013). However, a direct role of SEs in mediating plant stress responses has yet to be demonstrated, since the block of SE biosynthesis in the *erp1* mutant triggers a concomitant increase in the levels of SGs, which could be the true responsible for the altered defence response. In fact, it is becoming increasingly evident that SGs are actively involved in mediating adaptive plant responses to biotic and abiotic stress (Ferrer et al., 2017, and the references cited therein).

The synthesis of SEs is catalyzed by a group of enzymes collectively known as sterol acyltransferases. Depending on whether the acyl donor substrate is a long-chain fatty acyl-CoA or a phospolipid, sterol acyltransferases can be categorized into two main groups, namely, acyl-CoA:sterol acyltransferases (ASAT; EC 2.3.1.26) and phospholipid:sterol acyltransferases (PSAT; EC 2.3.1.43), respectively (Korber et al., 2017). Plant sterol acyltransferase activity has been reported to be primarily associated with membrane fractions in different species (Garcia and Mudd, 1978; Zimowski and Wojciechowski, 1981a,b; Kalinowska et al., 1989; Bouvier-Navé and Benveniste, 1995; Banas et al., 2005; Chen et al., 2007), but so far only ASAT and PSAT enzymes from Arabidopsis have been cloned and functionally characterized (Banas et al., 2005; Chen et al., 2007). The Arabidopsis ASAT1 (At3g51970) is structurally related to acyl-CoA sterol acyltransferases from yeast and animal systems and therefore has been included in the family of membrane-bound *O*-acyltransferases (MBOATs; Chang et al., 2011). Biochemical characterization of ASAT1 expressed in a mutant yeast strain lacking the two endogenous ASATs Are1p and Are2p revealed that the sterol precursor cycloartenol is the preferred acceptor of acyl groups. In line with this observation, overexpression of ASAT1 in Arabidopsis seeds resulted in the accumulation of cycloartenyl esters and to a lesser extent of 24-methylene cycloartenyl esters at the expense of campesteryl and β-sitosteryl esters and FSs, whose levels were substantially reduced (Chen et al., 2007). Arabidopsis PSAT1 (At1g04010) was also biochemically characterized using microsomal fractions obtained from leaves and roots of transgenic Arabidopsis overexpressing the enzyme. Phosphatidylethanolamine was the preferred acyl donor for sterol esterification and the enzyme was able to esterify several sterols and sterol intermediates. Despite sterol intermediates are poor substrates for PSAT1, they are preferentially used when sterol end products, particularly β-sitosterol, are present in the reaction mixture. The authors postulate an allosteric regulatory mechanism of PSAT1 activity by sterol end products that could modulate the amount of FSs in the membrane through a mechanism involving the sequestration of biosynthetic intermediates as SEs (Banas et al., 2005). More recently, the characterization of Arabidopsis T-DNA insertion mutants defective in the expression of *PSAT1* and *ASAT1* genes revealed a differential contribution of the corresponding enzymes to SE biosynthesis depending on the tissue. Thus, a 5- to 10-fold reduction of SE levels was observed in seeds of the *psat* mutants but not in those of the *asat* mutant, suggesting a major role of PSAT1 in the synthesis of SEs in seeds. On the contrary, SE levels were found to decrease in the leaves of both mutants. In spite of this, a phenotype of premature senescence associated to decreased SE levels was observed only in leaves of the *psat* mutant lines, which supports the notion that PSAT1 plays an important role in the maintenance of leaf viability during aging. Interestingly, the fact that SE levels in the flowers of both *psat* and *asat* mutants are similar to those of wild type plants (Bouvier-Navé et al., 2010) suggests that Arabidopsis may have an additional sterol acyltransferase that remains to be identified. The spatial localization of SE biosynthesis is another issue that requires further clarification. Arabidopsis ASAT1 is predicted to be an integral membrane protein (Chen et al., 2007), which is consistent with the reported detection of sterol acyltransferase activity in microsomal fractions from different tissues and plant species (Garcia and Mudd, 1978; Zimowski and Wojciechowski, 1981a,b; Kalinowska et al., 1989; Bouvier-Navé and Benveniste, 1995; Banas et al., 2005; Chen et al., 2007) and from yeast cells expressing the plant enzyme (Chen et al., 2007). Similarly, the activity of Arabidopsis PSAT1 has been detected in microsomal fractions from Arabidopsis plants overexpressing the enzyme (Banas et al., 2005). However, a more recent report has shown that a green fluorescent protein (GFP)-tagged version of Arabidopsis PSAT1 expressed in Arabidopsis and *Nicotiana benthamiana* plants localizes exclusively to cytoplasmic spherical structures of unknown identity that are believed to be neither LDs nor peroxisomes nor Golgi vesicles (Kopischke et al., 2013), which still leaves open the question regarding the true subcellular localization of this enzyme.

Tomato is one of the most important horticultural crops worldwide, and a well-established model plant for research in fleshy fruit development and ripening (Giovannoni, 2004), two biological processes in which SEs appear to play an important role (Whitaker, 1988). Some recent evidence also suggests that SEs might be involved in mediating plant defense responses against pathogen attack (Kopischke et al., 2013). All these observations prompted us to undertake this study aimed at the identification and characterization of the PSAT and ASAT enzymes of the dwarf tomato variety *Solanum lycopersicum* cv. Micro-Tom, as a first step toward the elucidation of the biological role of SE biosynthesis in tomato growth and development as well as in the adaptation to stress conditions.

## Materials and Methods

### Plant Material, Growth Conditions, and Treatments

*Solanum lycopersicum* cv. Micro-Tom plants were grown in pots filled with a mixture of peat (Klasmann TS2), vermiculite, and perlite (2:1:1, v/v/v) at 25°C in a greenhouse. To grow seedlings under axenic conditions, seeds were sterilized as described by Ramírez-Estrada et al. (2017) and sown in glass jars containing 0.5× Murashige and Skoog (MS) basal salts medium (pH 5.8) supplemented with Gamborg B5 vitamins and sucrose 3% (w/v), and solidified with 0.8% (w/v) plant agar. Jars were kept in darkness at 24°C for 2 days and transferred to a growth chamber under a 16 h light/8 h dark illumination regime (150 μE m^-2^s^-1^ intensity) at 24°C. For treatments with different compounds, after 12 days, pools of five seedlings were transferred to glass jars containing 30 mL of previously described MS liquid medium without sucrose and allowed to grow under the same conditions. After 1 week, the growth medium was replaced by fresh medium supplemented with the different compounds tested: 200 mM mannitol, 150 mM NaCl, 0.1 mM abscisic acid (ABA), 0.5 mM salycilic acid (SA), 0.5 mM methyl jasmonate (MeJ), or 1 μM flagellin 22. For cold treatment, seedlings were transferred to a growth chamber set at 4^o^C. Seedlings were sampled at different time points (0, 3, 6, 12, 24, and 48 h) and stored frozen at -80°C for further utilization.

*Arabidopsis thaliana* plants used throughout this study were of the Columbia-0 ecotype. The *psat1-2* and *asat1-1* mutant lines (Bouvier-Navé et al., 2010) were obtained from the Nottingham Arabidopsis Stock Center (NASC). For growth in soil, seeds were sown in pots containing a mixture of soil, perlite, and vermiculite (1:1:1, v/v/v). To obtain sterile seedlings, surface sterilized seeds were sown on plates containing half-strength MS medium supplemented with 1% (w/v) sucrose and solidified with 0.8% (w/v) plant agar. All seeds were stratified for 3 days at 4°C in the dark. Seedlings were grown for 15 days in a chamber set at 22°C under a 16 h light/8 h dark (long day) illumination regime (100 μE m^-2^ s^-1^) whereas adult plants were usually grown under a 8 h light/16 h dark (short day) illumination regime.

Mevalonate treatment of Arabidopsis wild type, *psat1-2*, and *psat1-2Pro35S::SlPSAT1* lines was performed essentially as described by Bouvier-Navé et al. (2010). Two-week-old seedlings grown on MS plates as described above were transferred into beakers containing liquid one-half-strength MS salts and 1% sucrose (w/v; 10 seedlings in 100 mL). Mevalonate was added to the medium as a 5 M stock solution (Nieto et al., 2009) to obtain a 3 mM final concentration. After 2 more weeks growing under the same temperature and light conditions with constant shaking at 120 rpm, seedlings were collected and imaged with a Nikon D7000 digital camera.

For leaf senescence analysis, fully expanded rosette leaves of plants grown for 6 weeks under short day conditions were detached of wild type, *psat1-2*, and *psat1-2Pro35S::SlPSAT1* plants. Leaves were placed in Petri dishes containing deionized water and incubated at 24°C under long day light conditions. Pictures were taken after 2 weeks with a Nikon D7000 digital camera.

### Cloning of SlPSAT1 and SlASAT1 cDNA Sequences

The coding regions of AtASAT1, SlASAT1, and SlPSAT1 were amplified by PCR using high-fidelity AccuPrime Taq DNA polymerase (Invitrogen), specific primer pairs encompassing the corresponding start and stop codons (Supplementary Table 1), and cDNA synthesized from total RNA obtained from *A. thaliana* leaves or tomato red fruit pericarp. RNA isolation and cDNA synthesis were performed as described previously (Ramírez-Estrada et al., 2017). The CACC sequence was added to the 5′ end of the forward primers to facilitate directional insertion of the amplified sequences into the pENTR/D-TOPO vector by Gateway recombination-based cloning (TOPO cloning kit, Invitrogen). The cDNAs in the resulting pENTR-AtASAT1, pENTR-SlASAT1, and pENTR-SlPSAT1 plasmids were sequenced to exclude the presence of amplification mutations.

### Arabidopsis Plant Transformation

The Gateway recombination system (LR clonase enzyme, Invitrogen) was used to transfer the SlPSAT1 and SlASAT1 cDNAs from the pENTR-SlPSAT1 and pENTR-SlASAT1 plasmids to the plant expression binary vector pEarleyGate100 (Earley et al., 2006), which contains a Basta^TM^ selectable marker and was obtained from the Arabidopsis Biological Resource Center (ABRC; stock CD3-724). In both cases, the coding sequences were cloned under the control of the cauliflower mosaic virus promoter (*CaMV35S*). The resulting constructs pEG100-SlPSAT1 and pEG100-SlASAT1 were introduced into *Agrobacterium tumefaciens* GV3101 via electroporation and used for transformation of the Arabidopsis mutants *psat1-2* and *asat1-1*, respectively, by the floral dip method (Clough and Bent, 1998). Seeds from transgenic plants resistant to the Basta^TM^ herbicide were analyzed for Basta^TM^ resistance segregation, and those from plants with a 3:1 segregation ratio were grown to obtain T_3_ homozygous lines. Expression of SlPSAT1 or SlASAT1 mRNA in the two transgenic lines selected for further characterization (*psat1-2Pro35S::SlPSAT1* and *asat1-1Pro35S::SlASAT1*) was checked by semiquantitative RT-PCR using *SlASAT1* and *SlPSAT1* gene specific primers (Supplementary Table 1).

### RT-qPCR Analysis of *SlPSAT1* and *SlASAT1* Gene Expression

The cDNA samples for RT-qPCR gene expression analysis were prepared from DNA-free total RNA samples obtained as indicated above. Real-time PCR assays were performed in triplicate with SYBR Green I Master (Roche Diagnostics) using a Light Cycler 480 detection system (Roche Diagnostics), essentially as described by Ramírez-Estrada et al. (2017). Specific primer pairs were designed for *SlPSAT1* and *SlASAT1* (Supplementary Table 1). The amount of target mRNA was normalized by using the tomato clathrin adaptor complexes (*CAC*) medium subunit gene (Solyc08g006960) as housekeeping reference gene. High-throughput RT-qPCR analyses of *SlPSAT1* and *SlASAT1* gene expression in response to different effectors were performed as previously reported (Ramírez-Estrada et al., 2017) using RNA samples obtained from tomato seedlings grown in MS liquid medium and treated as indicated above, and primers described in Supplementary Table 1.

### Subcellular Localization of SlPSAT1, SlASAT1, and AtASAT1 Proteins

The AtASAT1, SlASAT1, and SlPSAT1 coding sequences lacking the stop codon were amplified using AccuPrime Taq DNA polymerase, the respective pENTR plasmids as template and specific primer pairs (Supplementary Table 1). The resulting PCR products were cloned into pENTR/D-TOPO vector and subsequently transferred to the C-terminal YFP or GFP binary fusion vectors pEarleyGate101 and pEarleyGate103 (Earley et al., 2006; ABRC stocks CD3-683 and CD3-685), to generate clones encoding SlASAT1-YFP, AtASAT1-YFP, and SlPSAT1-GFP fusion proteins, respectively. All constructs were sequenced to confirm the in-frame fusions. In all cases, the coding sequences were under the control of the *CaMV35S* gene promoter. Recombinant plasmids coding for the tomato and Arabidopsis fusion proteins were transformed by electroporation into *A. tumefaciens* strain C58C1 (pGv2260). The resulting strains were separately mixed in a 1:1 ratio with an *A. tumefaciens* strain harboring the HC-Pro silencing suppressor (Goytia et al., 2006) and infiltrated in leaves of 3–4-week-old *N. benthamiana* plants. Plants were kept growing under long day conditions at 25°C and after 3 days, leaves were also infiltrated with either a Nile Red solution (1 mg/mL) to stain lipid bodies or a propidium iodide (PI) solution (5 mg/mL) to stain the cell wall. The abaxial epidermis of pieces of agroinfiltrated leaf tissue was scanned with an Olympus FV1000 confocal microscope (Tokyo, Japan) using the 60× water-immersion NA:1.20 objective. The 488-nm argon ion laser was used to excite YFP, GFP, and Nile Red, and the 599-nm diode laser was used for PI excitation. The emission windows for visualization of fluorescence were set at 500–545 nm for YFP and GFP and at 570–670 nm for Nile Red and PI. FV10-ASW software (Olympus) was used for image capture and ImageJ-32^1^ for merging false-colored images of transiently co-transformed cells. Fluorescence recovery after photobleaching (FRAP) analysis and fractionation of agroinfiltrated *N. benthamiana* leaf tissue into membrane and soluble fractions were performed as previously described (Ramírez-Estrada et al., 2017). To prevent aggregation of membrane proteins upon boiling, samples for immunoblot analysis were incubated at 37°C for 10 min (Kaur and Bachhawat, 2009) prior to subjecting the denatured protein samples to 10% SDS–polyacrylamide gel electrophoresis (SDS–PAGE; Laemmli, 1970).

### Sterol Analysis

Approximately 100 mg of seeds or 1 g of seedlings and rosette leaves was frozen in liquid nitrogen, grinded to a fine powder, and lyophilized. For sterol extraction, samples were suspended in 3 mL of a chloroform-methanol (2:1) mixture containing 10 μg of cholestanol (Sigma–Aldrich) and 10 μg of cholestanyl palmitate synthetized as described in Supplementary Material, as internal standards. After vigorous vortexing and sonication for 10 min in an ultrasonic water bath at room temperature, 1.5 mL of 0.9% (w/v) NaCl was added to improve phase separation. The organic phase was recovered by centrifugation at 3,000 × *g* for 5 min at room temperature and transferred to a new tube. The remaining aqueous phase was re-extracted with 3 mL of chloroform-methanol (2:1) and the two organic extracts were mixed and evaporated to dryness. The dried residue was dissolved in 100 μL of chloroform-methanol (2:1) and the FE and SE fractions were separated by TLC using precoated silica gel PLC 60 F254 plates (20 cm × 20 cm; Merck, Darmstadt) and dichloromethane as a mobile phase. Free cholestanol and cholestanyl palmitate standards were also applied onto the TLC plates. Plates were sprayed with a 0.01% primuline (Sigma-Aldrich) solution. FS and SE bands were visualized with an UV lamp and scraped from the silica plates for further sterol extraction. The SE fraction was saponified in 1.5 mL of 7.5% KOH methanolic solution. After incubation at 85°C for 2 h, the reaction was quenched with 1 mL of 0.9% (w/v) NaCl. FSs released from SEs and FSs scraped from the silica plates were extracted twice with 3 mL of *n*-hexane. The hexanic phases were collected by centrifugation, mixed, and evaporated to dryness. Sterols were derivatized by adding 50 μL of Bis(trimethylsilyl) trifluoroacetamide (BSTFA; Regis technologies) followed by a 2-h incubation at 80°C. After evaporation to dryness, sterols were dissolved in 100 μL of isooctane and analyzed by GC-MS, using an Agilent 7890A gas chromatograph equipped with a TEKNOKROMA TR-450232 capillary column (30 m × 0.25 mm × 0.25 mm) and coupled with a 5975C mass spectrometer (Agilent). Quantification of sterols was based on the relative peak area of cholestanol.

## Results

### Identification and Cloning of Candidate Genes Encoding Tomato Sterol Acyltransferases

**FIGURE 1:**
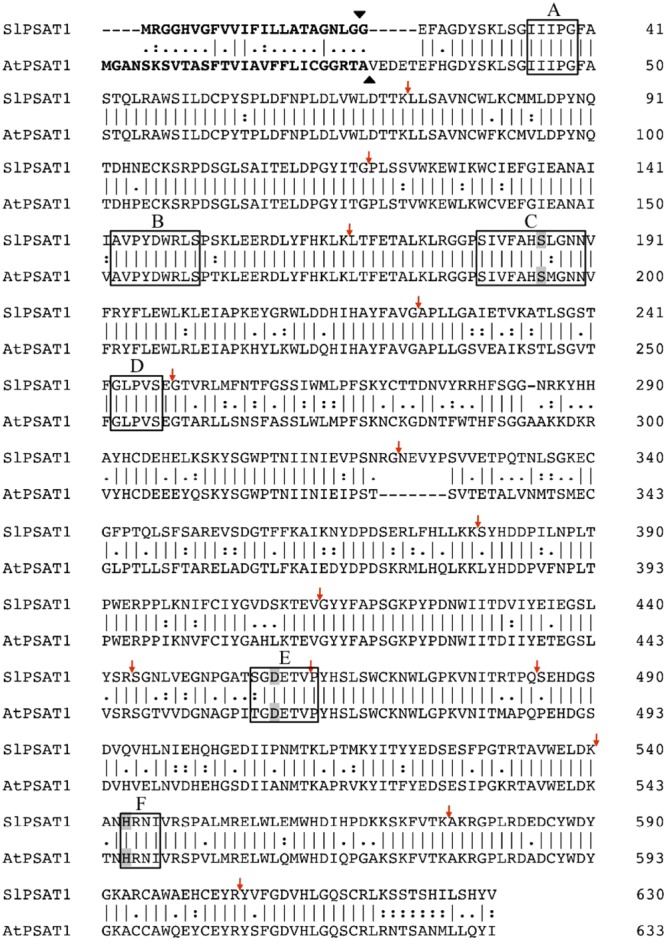
Sequence alignment of *S. lycopersicum* cv. Micro-Tom and *A. thaliana* PSAT1 proteins. Amino acid sequences were aligned using the Clustal Omega multiple sequence alignment tool (http://www.ebi.ac.uk/Tools/msa/clustalo/). Amino acid residues are numbered on the right. Vertical hyphens denote residues conserved in the two sequences. Colons indicate conservation between amino acids of strongly similar properties whereas periods indicate conservation between amino acids of weakly similar properties. Hyphens indicate gaps introduced to optimize the alignment. Vertical arrows denote positions at which introns interrupt the PSAT1 amino acid sequences. The six conserved regions in LCAT-like proteins (A–F) are boxed and amino acid residues of the catalytic triad (Ser–Asp–His) in conserved regions C, E, and F, respectively (Banas et al., 2005) are shaded. The predicted N-terminal signal peptides are shown in bold and the putative cleavage sites are shown by black arrowheads. The sequences shown have the following GenBank accession numbers: MG865284 (SlPSAT1) and AY989885 (AtPSAT1).

**FIGURE 2:**
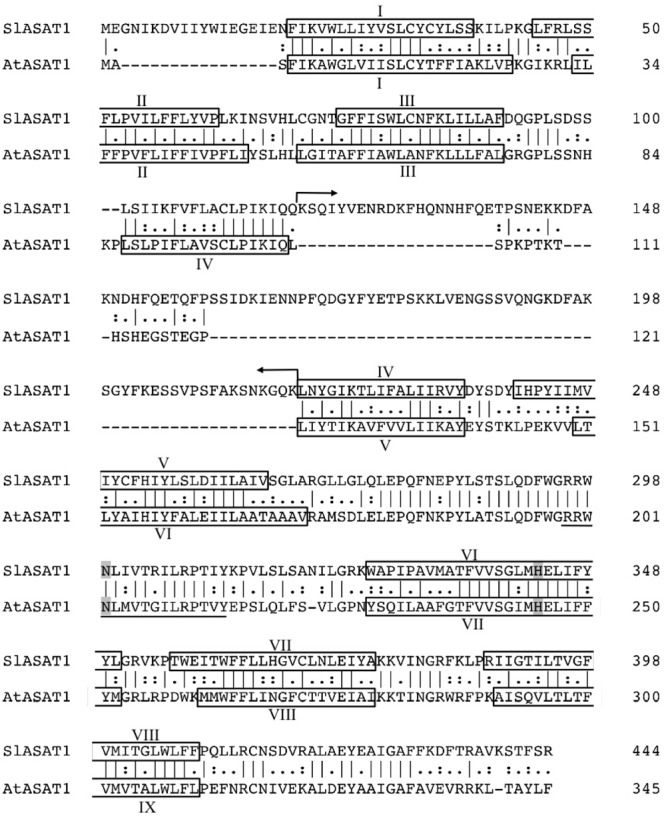
Sequence alignment of *S. lycopersicum* cv. Micro-Tom and *A. thaliana* ASAT1 proteins. Amino acid sequences were aligned using the Clustal Omega multiple sequence alignment tool (http://www.ebi.ac.uk/Tools/msa/clustalo/). Amino acid residues are numbered on the right. Vertical hyphens denote residues conserved in the two sequences. Colons indicate conservation between amino acids of strongly similar properties whereas periods indicate conservation between amino acids of weakly similar properties. Hyphens indicate gaps introduced to optimize the alignment. Arrows delimit the 100 amino acid stretch insertion in SlASAT1. The predicted transmembrane sequences I–VIII in SlASAT1 and I–IX in AtASAT1 are boxed. The catalytic amino acid residues (Asn and His) located in a conserved hydrophylic region (underlined) and the predicted VI and VII transmembrane domains, respectively, are shaded. The sequences shown have the following GenBank accession numbers: MG865283 (SlASAT1) and AAQ65159 (AtASAT1).

A search in the Phytozome database^2^ using as queries the amino acid sequence of the Arabidopsis sterol acyltransferases PSAT1 (Banas et al., 2005) and ASAT1 (Chen et al., 2007) retrieved a single PSAT (further referred to as SlPSAT1) and eight putative ASAT tomato candidates (**Table 1**). The predicted SlPSAT1 consists of 630 amino acid residues with an overall identity of 75% with AtPSAT1 (**Figure 1**), whereas the predicted SlASAT candidates range in size from 317 to 444 amino acid residues (**Table 1**) and have overall identity values with AtASAT1 in the range between 33 and 49% (Supplementary Table 2). As a first step toward studying the synthesis of SEs in tomato, we selected for further characterization SlPSAT1 (Solyc09g072710) and the SlASAT candidate most closely related to AtASAT1 (49% overall identity; Supplementary Table 2 and **Figure 2**), which is, moreover, encoded by the most actively and widely expressed member of the *SlASAT* gene family (Solyc11g012260) according to the RNA-seq expression data available via the tomato eFP browser at bar.utoronto.ca (Supplementary Figure 1).

**Table 1:** List of tomato genes coding for SlPSAT and SlASAT candidates identified in the Phytozome database using as query the amino acid sequence of the corresponding Arabidopsis sterol acyltransferases.

PSAT gene ID	Amino acids
At1g04010	633
Solyc09g072710	630

**ASAT gene ID**	**Amino acids**

At3g51970	345
Solyc11g012260	444
Solyc11g012210	353
Solyc11g012230	353
Solyc11g012250	350
Solyc11g012240	353
Solyc11g012200	353
Solyc11g012220	355
Solyc12g089050	317

*The number of amino acids of candidate proteins is shown in the right column.*

To this end, we amplified the SlPSAT1 and SlASAT1 ORFs by RT-PCR from RNA of tomato (*S. lycopersicum* cv. Micro-Tom) pericarp tissue using specific primer pairs (Supplementary Table 1). Alignment of the cloned cDNAs with the corresponding genomic sequences in the Phytozome database revealed that *SlPSAT1* consists of 15 exons separated by 14 introns (**Figure 1**) whereas *SlASAT1* has no introns, like their Arabidopsis counterparts. Conceptual translation of the cDNA sequences confirmed that the amino acid sequences of SlPSAT1 (630 amino acids) and SlASAT1 (444 amino acids) were identical to those found in the Phytozome database, and also that SlPSAT1 is three amino acids shorter than AtPSAT1 (**Figure 1**) whereas SlASAT1 is substantially longer than AtASAT1, mainly due to a long amino acid stretch of 100 residues comprised between positions 119 and 218 (**Figure 2**). The analysis of SlPSAT1 amino acid sequence with Signal P predicts the occurrence of an N-terminal signal peptide of 23 amino acids in length (Supplementary Table 3) that is also found in AtPSAT1 (**Figure 1**). The tomato enzyme also possesses six conserved regions and the catalytic triad (Ser186–Asp458–His543; **Figure 1**) typical of PSAT proteins from different species, including mammalian lecitin:cholesterol acyl transferases (LCAT) and AtPSAT1 (Banas et al., 2005). On the contrary, the amino acid sequence of SlASAT1 is predicted to contain eight transmembrane sequences according to the Protter 1.0 program (Omasits et al., 2014) as well as the two key amino acid residues reportedly important for catalysis (Asn299 and His343; **Figure 2**) found in all MBOATs (Hofmann, 2000; Huang et al., 2014).

### Functional Characterization of Tomato PSAT1 and ASAT1

**FIGURE 3:**
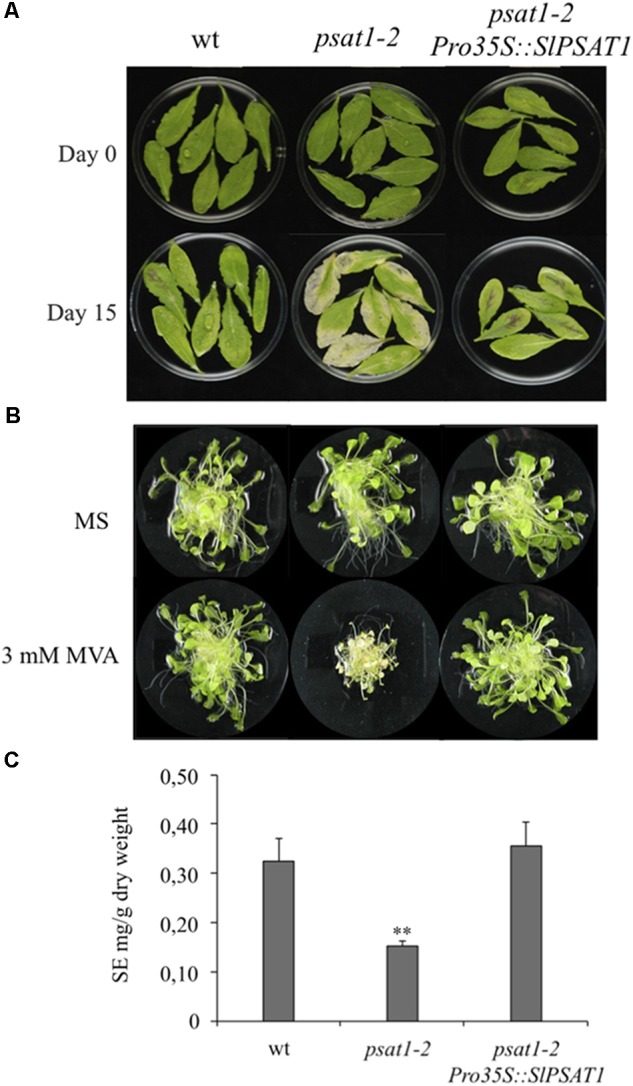
Tomato PSAT1 complements the morphological and biochemical phenotypes of *psat1-2* mutant. **(A)** Fully expanded green rosette leaves of the same age were detached from wild type, *psat1-2* mutant, and *psat1-2* mutant plants overexpressing SlPSAT1 (*psat1-2Pro35S::SlPSAT1*) grown for 6 weeks under long day conditions (16 h light/8 h darkness) and incubated in water for 15 days under the same light conditions. **(B)** Arabidopsis wild type, *psat1-2*, and *psat1-2* overexpressing SlPSAT1 (*psat1-2Pro35S::SlPSAT1*) plants were grown on solid MS medium and after 15 days transferred to liquid MS medium with or without 3 mM MVA. Images of plantlets were taken 15 days after transfer to liquid medium. **(C)** Content of sterified sterols (SE) in rosette leaves of Arabidopsis wild type, *psat1-2* mutant, and *psat1-2* overexpressing SlPSAT1 (*psat1-2Pro35S::SlPSAT1*). Total SE content includes sterified cholesterol, campesterol, stigmasterol, and β-sitosterol, and was determined as described in section “Material and Methods.” Values are means ± *SD* (*n* = 3). Asterisks show the values that are significantly different (^∗∗^*p* < 0.01) compared to those in wild type leaf samples.

In order to verify the functional identity of SlPSAT1, we checked its capacity to complement the morphological and biochemical phenotypes of the Arabidopsis *psat1-2* knock-out mutant (Bouvier-Navé et al., 2010). Constitutive expression of SlPSAT1 (Supplementary Figure S2) fully reversed the early senescence phenotype observed in detached rosette leaves of *psat1-2* (**Figure 3A**) and restored its capacity to grow in the presence of exogenously supplied MVA (**Figure 3B**). The addition of MVA to the growth medium leads to enhanced levels of FSs that become highly toxic in the absence of a functional PSAT (Bouvier-Navé et al., 2010). In line with the above, expression of SlPSAT1 also restored wild type SE levels in leaves of the *psat1-2* mutant, which are reduced below 50% compared to the wild type plants (**Figure 3C**). Overexpression of SlPSAT1 did not cause significant changes on total FS content in the retransformed *psat1-2* plants compared with untransformed controls (Supplementary Figure 3). Overall, these results confirmed that SlPSAT1 is the actual tomato ortholog of AtPSAT1.

**FIGURE 4:**
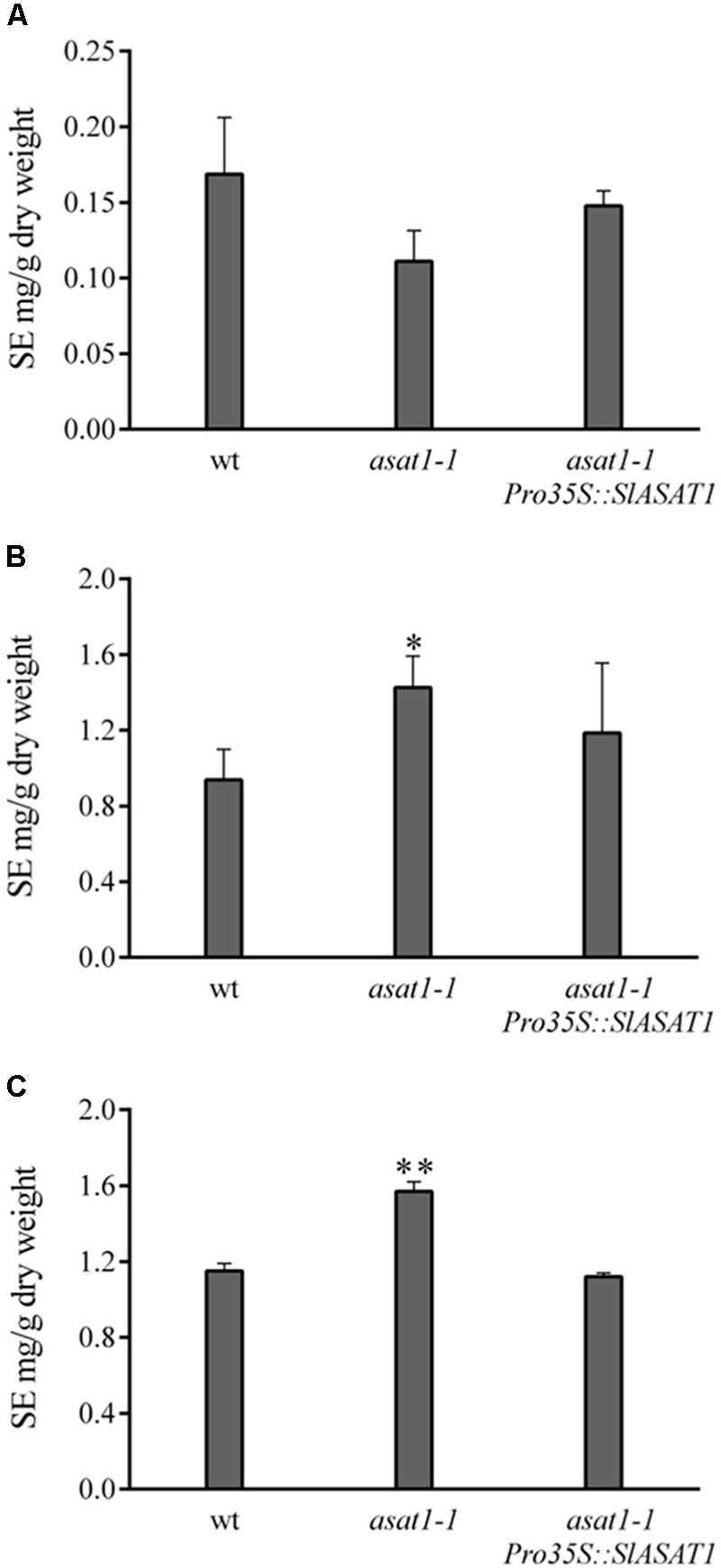
Content of esterified sterols (SE) in rosette leaves **(A)**, seedlings **(B)**, and seeds **(C)** of Arabidopsis wild type, *asat1-1* mutant, and *asat1-1* overexpressing SlASAT1 (*asat1-1Pro35S::SlASAT1*). Total SE content includes esterified cholesterol, campesterol, stigmasterol, β-sitosterol, isofucosterol, cycloartenol, and 24-methylene cycloartanol, and was determined as described in section “Material and Methods.” Values are means ±*SD* (*n* = 3). Asterisks show the values that are significantly different (^∗^*p* < 0.05 and ^∗∗^*p* < 0.01) compared to those in the corresponding wild type tissue samples.

**FIGURE 5:**
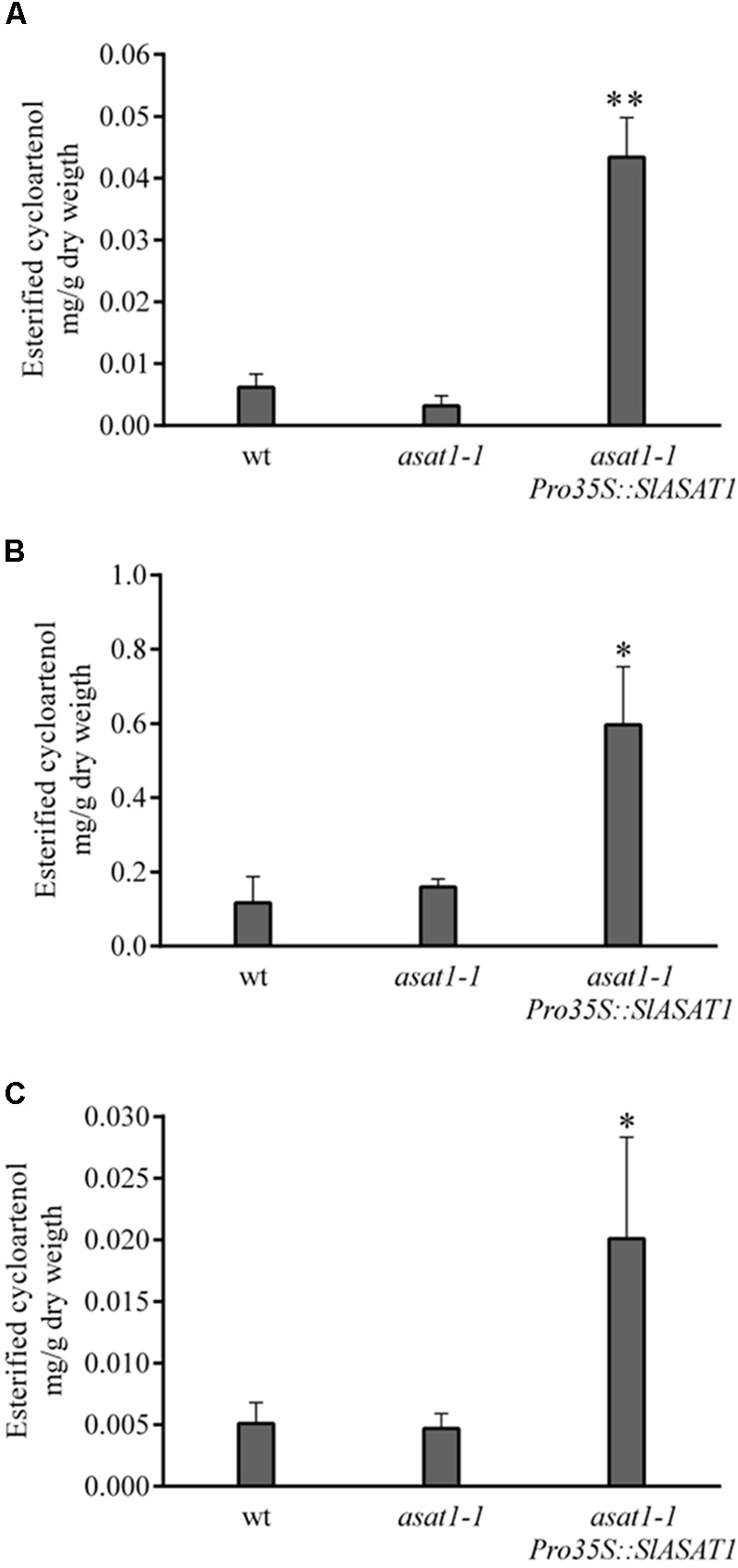
Content of esterified cycloartenol in rosette leaves **(A)**, seedlings **(B)**, and seeds **(C)** of Arabidopsis wild type, *asat1-1* mutant, and *asat1-1* overexpressing SlASAT1 (*asat1-1Pro35S::SlASAT1*). Values are means ±*SD* (*n* = 3). Asterisks show the values that are significantly different (^∗^*p* < 0.05 and ^∗∗^*p* < 0.01) compared to those in the corresponding wild type tissue samples.

We used a similar functional complementation approach to check the functionality of SlASAT1 taking advantage of the Arabidopsis *asat1-1* mutant. This mutant does not show any of the phenotypes displayed by the *psat1-2* mutant and does not display any obvious morphological difference when compared to the wild type, which likely reflects the moderate reduction of SE levels compared with that in *psat1-2* mutant. In fact, total SE levels in *asat1-1* rosette leaves were only reduced by about 30% (**Figure 4A**) as previously reported by Bouvier-Navé et al. (2010). Sterol analysis in other organs of *asat1-1* revealed, contrary to expectations, significantly increased SE levels in seeds and seedlings (about 36 and 50%, respectively) compared to the wild type (**Figures 4B,C**). Such an upward trend of SE content in seeds of the *asat1-1* mutant had been previously observed, although it was considered not to be significant (Bouvier-Navé et al., 2010). Either way, expression of SlASAT1 in the *asat1-1* background (Supplementary Figure 2) restored total SE content to wild type or near wild type levels in all tested tissue samples (**Figure 4**), thus indicating that SlASAT1 is a tomato ortholog of AtASAT1. Interestingly, a detailed analysis of the SE profile in these three samples (Supplementary Figure 4) revealed that SlASAT1 expression drastically enhanced the content of esterified cycloartenol (fourfold, fivefold, and sevenfold in seeds, seedlings, and leaves, respectively) compared to wild type samples (**Figure 5**), and to a much lesser extent that of esterified 24-methylene cycloartanol. The content of this compound increased in seedlings expressing SlASAT1 compared to the wild type and the *asat1-1* mutant, and became detectable in seeds and leaves. Esterified 24-methylene cycloartanol in these tissues of wild type and *asat1-1* mutant plants was below detection limit (Supplementary Figure 4). These results indicate that SlASAT1 has a clear substrate preference for the sterol precursor cycloartenol, just like AtASAT1, and moreover that the lack of AtASAT1 triggers an upregulation of SE biosynthesis in Arabidopsis seeds and seedlings, but not in leaves. Nor were changes observed in the total content of FSs in seedlings and seeds of the *asat1-1* mutant, whereas a modest increase was observed in leaves (Supplementary Table 4). Altogether, these results demonstrate that the selected SlASAT1 candidate is a functional homolog of AtASAT1 and suggest that plant sterol homeostasis is differentially regulated depending on the plant tissue.

### Expression Analysis of *SlPSAT1* and *SlASAT1* Genes

**FIGURE 6:**
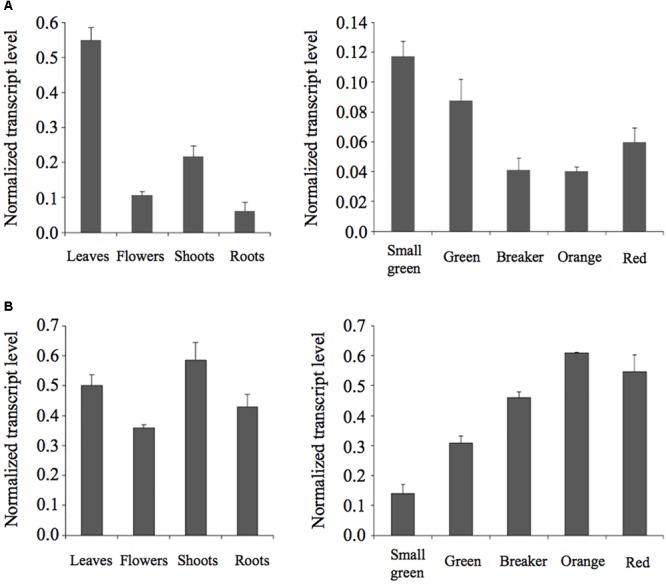
Expression analysis of *SlPSAT1* and *SlASAT1*. The mRNA levels of *SlPSAT1* (upper panels) and *SlASAT1* (lower panels) were determined by RT-qPCR in RNA samples obtained from tomato leaf, flower, shoot, and root tissue **(A)**, and fruits at the indicated developmental and ripening stages **(B)**. Transcript levels were normalized relative to the mRNA levels of the clathrin adaptor complexes (CAC) medium subunit gene (Solyc08g006960). Values are means ±*SD* (*n* = 9).

We performed RT-qPCR analyses to investigate the expression of the *SlPSAT1* and *SlASAT1* genes in different organs of tomato plants and fruits at different stages of development and ripening. As shown in **Figure 6**, *SlPSAT1* and *SlASAT1* expression was detected in all tissues analyzed. *SlPSAT1* was actively expressed in leaves and to a much lesser extent in roots, flowers, and shoots (**Figure 6A**). On the contrary, similar levels of SlASAT1 mRNA were measured in all these tissues (**Figure 6B**). Interestingly, *SlPSAT1* and *SlASAT1* showed highly complementary expression patterns in fruits. The transcript levels of *SlPSAT1* decreased progressively when fruits developed and started to ripe (small green to orange stages) and increased slightly when ripening progressed (red mature stage). On the contrary, the expression of *SlASAT1* increased progressively throughout fruit development and ripening (small green to orange stages) and remained at similar high levels at the red mature stage. Overall, these results demonstrate that *SlPSAT1* and *SlASAT1* genes are differentially expressed in different organs of tomato plants as well as during fruit development and ripening.

### Subcellular Localization of SlPSAT1 and SlASAT1

**FIGURE 7:**
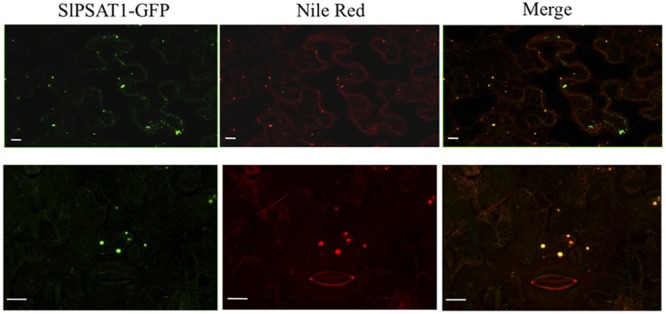
Confocal laser scanning micrographs showing the fluorescence pattern of *N. benthamiana* cells expressing SlPSAT1-GFP (left images) that were incubated with Nile Red prior to confocal analysis to stain lipid bodies (middle images). Co-localization of SlPSAT1-GFP and Nile Red fluorescence is shown in the corresponding merged images (right images). The boundaries of a closed stoma are visible in the Nile Red fluorescent images (bottom middle and right images). Scale bars = 10 μm.

To establish the subcellular localization of SlPSAT1, we transiently expressed in *N. benthamiana* leaves a C-terminal fusion of SlPSAT1 with the GFP and analyzed the resulting fluorescence pattern by confocal laser microscopy. SlPSAT1-GFP fluorescence was localized in cytoplasmic spherical structures that were also stained when leaves were incubated with Nile Red (**Figure 7**), a selective fluorescent dye suitable for staining cytoplasmic LDs containing neutral lipids like SEs (Greenspan et al., 1985). This result demonstrated that SlPSAT1-GFP localizes to LDs.

**FIGURE 8:**
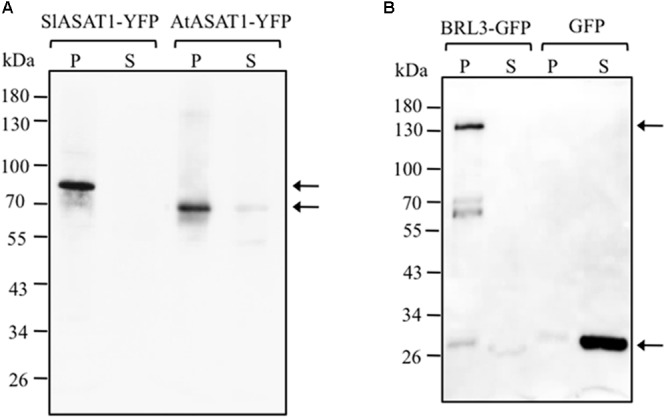
Immunoblot analysis of membrane (P) and soluble (S) cell fractions obtained from *N. benthamiana* leaf zones expressing SlASAT1-YFP and AtASAT1-YFP proteins **(A)** and BRL3-GFP and GFP proteins **(B)**, which were expressed as controls of plasma membrane- and cytosol-localized proteins, respectively. Arrows on the right side show the position of protein fusions whose predicted molecular weights are approximately 77.5 kDa (SlASAT1-YFP), 65.5 kDa (AtASAT1-YFP), and 153.0 kDa (BRL3-GFP). The position of protein molecular-weight standards is shown on the left side.

**FIGURE 9:**
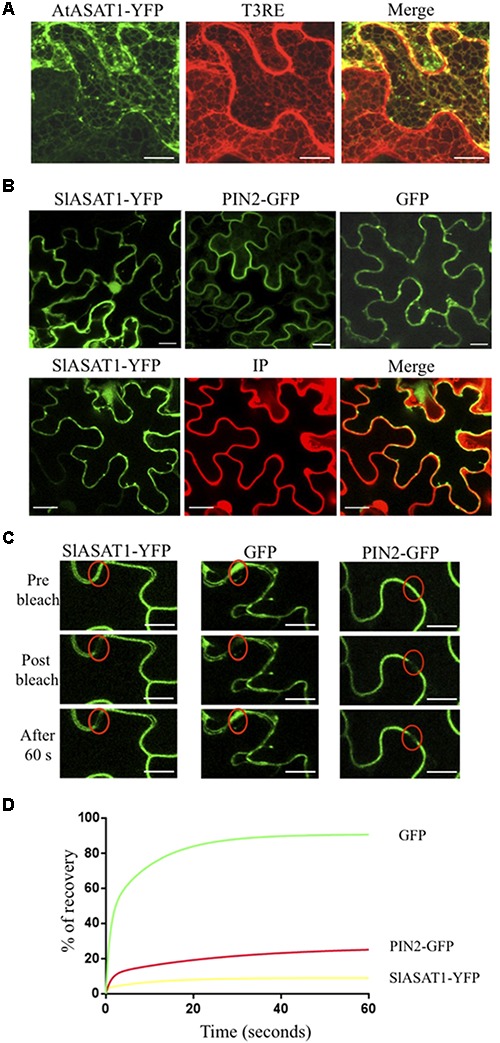
**(A)** Confocal laser scanning micrographs showing the fluorescence pattern of *N. benthamiana* cells co-expressing AtASAT1-YFP and the ER marker T3RE. Co-localization of AtASAT1-YFP and T3RE fluorescence is shown in the corresponding merged images. **(B)** Fluorescence pattern of cells expressing SlASAT1-YFP, PIN2-GFP, and GFP (upper panels). Cells expressing SlASAT1-YFP were stained with propidium iodide (PI) prior to confocal analysis to mark the cell wall (lower panels). Co-localization of SlASAT1-YFP and IP fluorescence is shown in the corresponding merged images. Scale bars = 20 μm. **(C)** FRAP analysis in *N. benthamiana* cells transiently expressing SlASAT1-YFP as well as GFP and PIN2-GFP proteins, which were expressed as cytosolic and PM control proteins, respectively. Regions of interest (ROIs) in cells expressing the fluorescent proteins (red ovals) were photobleached with a short pulse (1 s) of high-intensity laser light. The images shown were acquired just before photobleaching (left column), immediately after photobleching (middle column), and after 60 s of fluorescence recovery (right column). Scale bars = 10 μm. **(D)** FRAP curves representing the time course of SlASAT1-YFP, PIN2-GFP, and GFP fluorescence recovery between time points 0 and 60 s. Fluorescence recovery at the different time points is expressed as percentage of fluorescence at time point 0 s (pre-bleach). Fluorescence recovery curves represent the best fits from normalized datasets of 20 independently bleached ROIs (Supplementary Figure 5).

The analysis of the primary structure of SlASAT1 and AtASAT1 with the Protter 1.0 program (Omasits et al., 2014) predicted the occurrence of eight and nine transmembrane sequences, respectively (**Figure 2**), which strongly suggested membrane localization for both proteins. Interestingly, a subsequent *in silico* analysis of both proteins with the Predotar software predicted AtASAT1 to localize in the endoplasmic reticulum (ER) with a probability of 99% whereas, unexpectedly, SlASAT1 was predicted to be a non-ER protein with a probability of 96% (Supplementary Table 5). In order to experimentally determine the subcellular localization of SlASAT1 and AtASAT1, we first performed immunoblot analysis of membrane and soluble cell fractions obtained from agroinfiltrated *N. benthamiana* leaves expressing C-terminal fusions of ASAT1 with YFP (AtASAT1-YFP and SlASAT1-YFP). The PM-bound brassinosteroid receptor BRL3 fused to the GFP (BRL3-GFP) and the cytosolic GFP were also expressed as control proteins. In agreement with the bioinformatic prediction, both SlASAT1-YFP and AtASAT1-YFP were detected in the membrane fraction, just like control BRL3-GFP, whereas, as expected, GFP was detected in the soluble fraction (**Figure 8**). To assess in more detail the subcellular localization of the YFP-tagged ASAT1 proteins, we also analyzed by confocal microscopy the fluorescence pattern of *N. benthamiana* cells expressing SlASAT1-YFP and AtASAT1-YFP. In accordance with the *in silico* prediction, expression of AtASAT1-YFP resulted in a reticulate pattern of green fluorescence that fully overlapped with the red fluorescence of the ER marker protein T3RE (Forés et al., 2006) when both proteins were co-expressed (**Figure 9A**), thus confirming the ER localization of AtASAT1-YFP. On the contrary, the fluorescence emitted by SlASAT1-YFP localized at the periphery of the cell, a pattern similar to that observed in cells expressing the membrane-localized auxin carrier protein PIN2 fused to the GFP (PIN2-GFP). Moreover, the localization of SlASAT1-YFP overlapped with the fluorescence of PI when cells expressing SlASAT1 were stained with PI prior to confocal analysis to delimit the cell periphery (**Figure 9B**). The results of confocal microscopy analysis together with those of immunoblot analysis in fractionated cell extracts (**Figure 8**) clearly pointed toward a localization of SlASAT1 in the PM, which was confirmed by FRAP analysis, a technique that allows evaluation of the rate of protein mobility in living cells (Reits and Neefjes, 2001; Wu et al., 2006; Held et al., 2008; Ishikawa-Ankerhold et al., 2012). Selected regions of interest (ROIs) in cells expressing SlASAT1-YFP, PIN2-GFP, and GFP were irradiated with a short pulse (1 s) of high intensity laser light to irreversibly photobleach the fluorophore in the ROIs (**Figure 9C**). Then, recovery of fluorescence in the irradiated area due to the migration of the non-photobleached fusion proteins back to the bleached area was monitored at different time points over a 60-s period (**Figure 9D** and Supplementary Figure 5). Membrane proteins like PIN2-GFP are expected to replenish the bleached region much more slowly than cytosolic proteins like GFP, because they are embedded into the more viscous medium of cell membranes and therefore have a much more limited mobility than free cytosolic proteins. As shown in **Figure 9D**, less than 10% of initial fluorescence intensity was recovered in cells expressing SlASAT1-YFP after 60 s, a value that was even smaller than that in cells expressing the PM-bound protein PIN2-GFP, where fluorescence recovery after 60 s was around 20% of the initial intensity. As expected, the recovery of fluorescence in cells expressing the soluble cytosolic GFP was close to 90%. Taken together, the results of FRAP analysis confirmed the localization of SlASAT1-YFP in the PM, which is in sharp contrast to the ER-localization of AtASAT1.

### Transcriptional Profiling of *SlPSAT1* and *SlASAT1* Gene Expression in Response to External Stimuli

**FIGURE 10:**
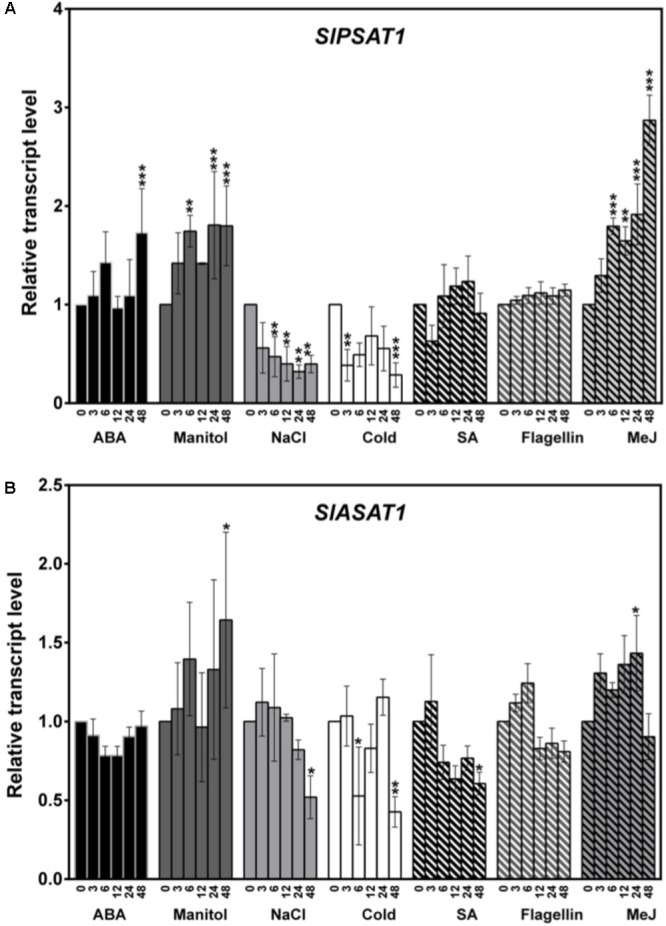
Expression of **(A)** *SlPSAT1* and **(B)** *SlASAT1* genes in tomato seedlings exposed to different stresses. The transcript levels were measured by RT-qPCR using RNA samples from tomato seedlings exposed to different treatments (ABA, manitol, NaCl, cold, SA, flagellin 22, and MeJ). Samples were collected at the indicated time points (3, 6, 12, 24, and 48 h) from the start of each treatment (0 h). Data are expressed as normalized quantity values calculated using two independent housekeeping genes (*PP2Acs* and *EF1a*; Ballester et al., 2013) and relative to non-treated seedlings at each time point, which is assumed to be one. Values are means ±*SD* (*n* = 6). Asterisks show the values that are significantly different (^∗^*p* < 0.05, ^∗∗^*p* < 0.01, and ^∗∗∗^*p* < 0.001) compared to those at time 0 h.

The expression profile of *SlPSAT1* and *SlASAT1* in response to different external stimuli, including the pathogen elicitor flagellin 22, the plant hormones ABA, MeJA and SA, and different stresses (osmotic, salt, and cold), was analyzed by RT-qPCR using RNA samples obtained from 3-week-old tomato seedlings collected before (0 h) and after different times of exposition to the mentioned stimuli (3, 6, 12, 24, and 48 h), and compared to that in non-treated seedlings collected at the same time points (**Figure 10**). The increase detected in the transcript levels of different marker genes reported as responsive to the assayed treatments in tomato confirmed the activation of the corresponding stress signaling pathways (Ramírez-Estrada et al., 2017).

In the case of *SlPSAT1*, the strongest induction was detected at time point 48 h after MeJ treatment started (about threefold higher than basal level). At this time point, the expression of *SlPSAT1* also increased significantly in response to osmotic stress and ABA treatment (about twofold). However, while the effect of ABA was only observed at time point 48 h, the induction by MeJ and manitol was already observed at 6 h post-treatment and was maintained until the end of the treatment (**Figure 10A**). By contrast, *SlPSAT1* expression was downregulated after 6 h of exposure to cold and salt stresses, and the transcript levels remained below those in the non-treated plants until the end of the experiment (**Figure 10A**). The transcript levels of this gene were not significantly altered in plants exposed to SA and flagellin treatments (**Figure 10A**). The expression profile of *SlASAT1* in response to the assayed stimuli broadly paralleled that of *SlPSAT1*, although to a lower quantitative degree (**Figure 10B**). Indeed, *SlASAT1* expression was also induced upon manitol and MeJ treatments at time points 48 and 24 h, respectively, and decreased 48 h after salt, cold, and SA treatments, but these changes did not exceed twofold in any of the assayed stresses (**Figure 10B**). These results show that the expression of the two genes encoding tomato sterol acyltransferases is regulated by stress, although *SlPSAT1* seems to behave as a more stress-responsive gene than *SlASAT1*.

## Discussion

In this study, we report the identification, cloning and functional characterization of the tomato sterol acyltransferases SlPSAT1 and SlASAT1. The tomato genome contains a single gene that encodes the ortholog of AtPSAT1 (**Table 1**), as demonstrated by the ability of SlPSAT1 to complement both the morphological and biochemical phenotypes of the Arabidopsis *psat1-2* knock-out mutant (**Figure 3**) lacking PSAT1 activity (Bouvier-Navé et al., 2010). This is fully consistent with the fact that SlPSAT1 shares 75% sequence identity and the presence of an N-terminal signal peptide with AtPSAT1, and possesses the six conserved regions (A–F), including the catalytic triad formed by Ser186 (region C), Asp458 (region E), and His543 (region F; **Figure 1**), found in PSAT and PSAT-like proteins from other eukaryotic organisms (Banas et al., 2005). As previously observed when AtPSAT1 was overexpressed in different Arabidopsis and tobacco genetic backgrounds (Bouvier-Navé et al., 2010), overexpression of SlPSAT1 in the Arabidopsis *psat1-2* mutant restored wild type SE content but did not result in an increased accumulation of SE or reduced levels of FSs compared to wild type plants (**Figure 3** and Supplementary Figure 3), thus reinforcing the view that PSAT1 is not a rate-limiting enzyme in plant sterol metabolism.

In contrast to SlPSAT1, the tomato genome contains up to eight genes coding for ASAT-like proteins. Among these, gene candidate Solyc11g012260 was found to encode the largest protein of this family (444 amino acid residues; **Table 1**), which is, moreover, the one sharing the highest degree of identity with AtASAT1 (49% overall identity; Supplementary Table 1). Interestingly, this gene is located on chromosome 11 in tandem with other six gene candidates (Solyc11g012200 to Solyc11g012250) that encode a subfamily of highly conserved proteins (69–80% overall identity; Supplementary Table 1) that are very similar in length to AtASAT1 (350–355 vs. 345 amino acid residues; **Table 1**), in spite of which they show a lower level of identity with AtASAT1 than SlASAT1 (Supplementary Table 1). The remaining candidate gene (Solyc12g089050) is located in a different chromosome and codes for the shortest (317 amino acid residues; **Table 1**) and most distantly related member of the tomato ASAT-like protein family (31–34% overall identity; Supplementary Table 1). The above observations, along with the fact that Solyc11g012260 is the most actively and widely expressed member of this gene family according to expression data in the Heinz variety available via tomato eFP browser at bar.utoronto.ca (Supplementary Figure 1), prompted us to select SlASAT1 for further functional characterization. As in the case of SlPSAT1, this was addressed by taking advantage of the Arabidopsis mutant *asat1-1* lacking ASAT1 activity, although in this case, the mutant displays a wild type phenotype with only a moderate reduction of SE levels in leaves (Bouvier-Navé et al., 2010). A detailed analysis of SE content in different organs of the *asat1-1* mutant confirmed the effect of AtASAT1 inactivation on SE levels in leaves, but revealed an unexpected increase of total SEs in both seedlings and mature seeds (**Figure 4**). This indicates that AtASAT1 is involved in SE biosynthesis not only in leaves, as previously reported (Bouvier-Navé et al., 2010), but also in other tissues, although its contribution to the maintenance of sterol homeostasis appears to be clearly different. Regardless of this, expression of SlASAT1 restored wild type or near wild type levels of SEs in all three tissues (**Figure 4**) and, moreover, resulted in a drastic accumulation of esterified cycloartenol, and to a lesser extent of sterified 24-methylene cycloartanol, in these tissues (**Figure 5** and Supplementary Figure 4). These two minor sterols, which are the first cyclic precursors in the post-squalene portion of the plant sterol pathway (Schaller, 2003), are also the preferred sterol substrates of AtASAT1 (Chen et al., 2007). These findings demonstrate that SlASAT1 is a true ortholog of AtASAT1 and indicate that the occurrence of an specialized ASAT responsible for esterification of sterol precursors is not restricted to Arabidopsis, but rather seems to be a general feature of plant SE metabolism, although its biological significance is far from clear. It has been suggested that esterified cycloartenol could be a more suitable way to transport sterols than esterified sterol end products due to its better micellar solubility (Chen et al., 2007). It might also be speculated that cycloartenol esterification can help to modulate the flux through the sterol pathway by regulating the amount of free cycloartenol available for sterol end product biosynthesis. In fact, the interconversion of cyclic sterol biosynthetic intermediates between free and esterified forms has been suggested to play a role in regulating the rate of the post-squalene portion of the sterol pathway (Dyas and Goad, 1993; Banas et al., 2005). It is thus conceivable that the lack of AtASAT1 activity in seeds and seedlings of the *asat1-1* mutant may lead to enhanced levels of free cycloartenol due to defective esterification of this intermediate. This, in turn, would enhance the flux through the sterol pathway leading to a subsequent increase of SE formation to accommodate the resulting excess of sterol end products. The opposite effect of the *asat1-1* mutation on leaf SE content (**Figure 4**) suggests that such a regulatory mechanism would operate only in certain tissues, as for instance those accumulating high levels of SEs.

The spatial organization of sterol esterification has not yet been fully elucidated. Sterol acyltrasferase activity has been found associated with microsomal membranes in different tissues and plant species (Garcia and Mudd, 1978; Zimowski and Wojciechowski, 1981a,b; Kalinowska et al., 1989; Bouvier-Navé and Benveniste, 1995; Banas et al., 2005; Chen et al., 2007). However, it has recently been reported that an AtPSAT1-YFP fusion expressed in Arabidopsis and *N. benthamiana* leaves localizes to cytoplasmic vesicular structures that were considered not to represent LDs (Kopischke et al., 2013). LDs are ubiquitous small cytoplasmic organelles that originate in the ER membrane and consist of a hydrophobic core, composed mostly of SEs and triacylglycerol, surrounded by a phospholipid monolayer that contains different proteins, some of which are involved in lipid metabolism (Wilfling et al., 2014; Welte and Gould, 2017). Our subcellular localization studies using a fluorescently tagged SlPSAT1 protein expressed in leaves of *N. benthamiana* revealed that SlPSAT1-GFP localizes in small cytoplasmic droplets that were also stained with the lipid specific dye Nile Red, thus indicating that under our experimental conditions SlPSAT1 localizes to LDs (**Figure 7**). The reason for the differential localization of AtPSAT1 and SlPSAT1 remains so far unknown, but what seems clear is that neither of the two proteins are associated to the ER. This might explain the extremely low levels of PSAT activity reported in microsomal fractions from Arabidopsis leaves and roots (Banas et al., 2005). On the other hand, the localization of SlPSAT1 in LDs is consistent with the proposed role of these organelles in plant sterol homeostasis. In tissues like seeds and pollen that have the ability to accumulate metabolic reserves, it is accepted that the main role of LDs is to serve as organelles for storage of membrane lipid precursors and high-energy metabolites. In these tissues, LDs would be involved in translocation of sterols in the form of SEs from ER to other cell membranes, including the PM. On the contrary, in non-storage vegetative organs such as leaves, stems, and roots, LDs are much less abundant and their primary role appears to be the removal of potentially toxic sterols and fatty acids scavenged from damaged membranes during stresses or senescence for subsequent recycling (Huang, 2018). In both cases, the localization of SlPSAT1 in LDs would facilitate the rapid conversion of FSs into ESs, whether they are removed from disorganized cell membranes or are synthesized *de novo*.

The subcellular localization of SlASAT1 was investigated using a combination of immunoblot and confocal microscopy analyses. The results of immunoblot analysis of membrane and soluble fractions from *N. benthamiana* cells expressing an SlASAT1-YFP fusion indicated that SlASAT1-YFP is attached to cell membranes (**Figure 8**), which is in full agreement with the prediction of eight transmembrane sequences in the SlASAT1 protein (**Figure 2**). A more detailed analysis using fluorescence microscopy techniques, including FRAP analysis, revealed that the fluorescence observed at the periphery of cells expressing SlASAT1-YFP really came from a protein located in the PM (**Figure 9B**). FRAP curves showed that the intracellular mobility of SlASAT1 was even slower than that of PIN2-GFP (**Figures 9C,D**), a well-characterized PM-bound protein. Such an extremely low diffusion rate indicates that SlASAT1 is a deeply embedded integral PM protein. The finding that SlASAT1 localizes in the PM is in full agreement with the result of an *in silico* analysis performed with the Predotar software, which predicts with a 96% of confidence that SlASAT is a non-ER protein. Interestingly, this analysis also predicts with a confidence of 99% that AtASAT1 is an ER-resident protein (Supplementary Table 5). This, together with the fact that a detailed study of the subcellular localization of AtASAT1 is missing, led us to investigate also the localization of an AtASAT1-YFP fusion protein using similar experimental approaches. The results of immunoblot assays (**Figure 8**) and confocal microscopy analysis (**Figure 9A**) revealed that AtASAT1-YFP clearly localize in the ER membrane, in sharp contrast to the PM localization of SlASAT1. In spite of this, SlASAT1 is able to complement the biochemical phenotype of the Arabidopsis *asat1-1* mutant (**Figure 4**). This observation raises the question of how the PM-localized SlASAT1 may have access to cycloartenol, a sterol substrate that is most likely embedded in the ER membrane where sterol biosynthesis is widely accepted to occur (Benveniste, 2004; Schaller, 2004).

**FIGURE 11:**
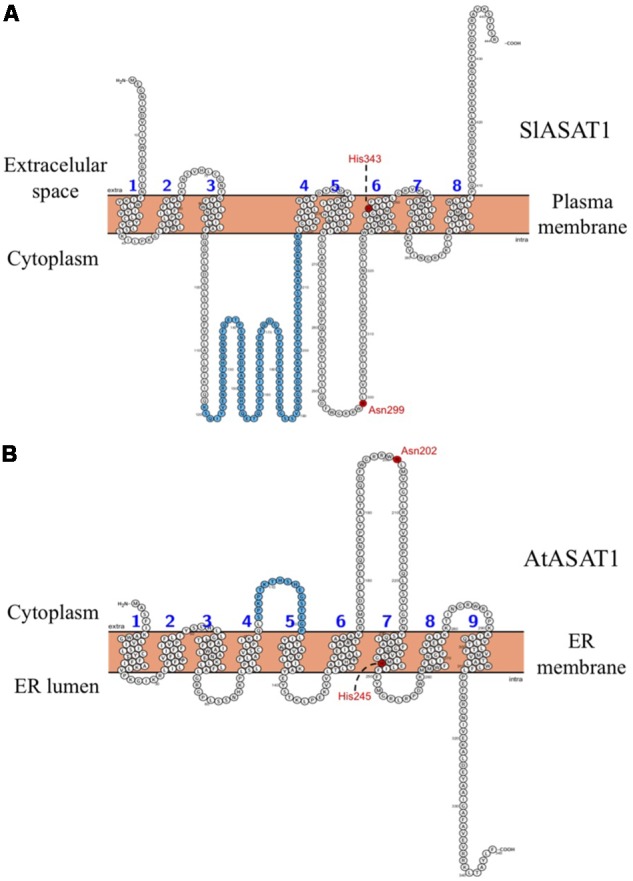
Membrane topology models of **(A)** SlASAT1 and **(B)** AtASAT1. Prediction was performed with the Protter 1.0 program (Omasits et al., 2014). The transmembrane sequences are numbered in blue. The Asn and His residues reportedly important for ASAT catalysis are shown in red. The sequence of 113 amino acid residues comprised between positions 119 and 218 in the SlASAT1 sequence, and the equivalent sequence of 17 amino acids comprised between positions 105 and 121 in the AtASAT1 sequence are shown in blue.

The possibility that enzymes located in one membrane can act on substrates present in a different cell membrane, the so-called *in trans* activity, is not unprecedented (Stefan et al., 2011; Haj et al., 2012; Mehrshahi et al., 2013; Tavassoli et al., 2013). In plants, like in other eukaryotic organisms, the ER membrane network is known to form physical contacts with the membrane of several other cell organelles, particularly with the PM at the cell periphery (Stefan et al., 2013; Pérez-Sancho et al., 2016). Thus, it is conceivable that SlASAT1 may act *in trans* on cycloartenol in the lipid bilayer at ER–PM contact sites to produce cycloartenyl esters that can be then packed in LDs for subsequent transport to other cell membranes or hydrolyzed back to its free form depending on the cell sterol biosynthetic needs. This hypothesis is compatible with the predicted arrangement of SlASAT1 in the PM, which shows that the catalytically important Asn residue (Asn299) would be located in a large cytoplasmic loop connecting transmembrane segments 5 and 6 (**Figure 11**). This residue is conserved in all members of the MBOAT family (Hofmann, 2000; Huang et al., 2014) and has been suggested to be involved in binding the long-chain fatty acyl-CoA utilized for sterol esterification (Chang et al., 2011). Interestingly, the predicted topology of AtASAT1 in the ER membrane shows that the equivalent Asn residue (Asn202) would be located in a similar cytosolic loop that connects transmembrane domains 6 and 7. In both enzymes, this loop is flanked on the C-terminal side by the transmembrane domain that contains the His residue reported also to be involved in enzyme catalysis (Lin et al., 2003). Thus, the predicted spatial orientation of SlASAT1 and AtASAT1 in their host membranes should enable SlASAT1 and AtASAT1 to access their substrate from either the PM or the ER, respectively. Interestingly, SlASAT1 has a second large cytosolic loop of 127 residues (from Asp92 to Lys218) that connects transmembrane domains 3 and 4. This loop is much longer than the equivalent loop in AtASAT1, which connects transmembrane domains 4 and 5, and encompasses only 18 amino acid residues (from Leu104 to Pro121; **Figure 11**). It can be speculated that this domain, which includes the additional 100 amino acid residues of SlASAT1 with regard to AtASAT1, might be somehow responsible for the different subcellular localization of SlASAT1 compared to AtASAT1 and/or contribute to its suggested *in trans* enzyme activity. Obviously, our results do not exclude the possibility that other members of the tomato ASAT-like enzyme family identified in this work (**Table 1**) might localize to the ER membrane where they could also participate in SE formation.

The experimental evidence supporting a direct involvement of SEs in plant stress response is scarce, in contrast to the many reports relating changes in the profile of free and glycosylated sterols with specific responses to different types of stress (Ferrer et al., 2017; Ramírez-Estrada et al., 2017). This likely reflects the fact that FSs, SGs, and ASGs are essential components of PM whereas SEs are a reservoir of FSs that do not localize in the PM but in LDs. Our gene expression results showed that SlPSAT1 transcript levels increase upon MeJ, mannitol, and, to a lower degree, ABA treatments, and decrease after exposure to salt or cold stress (**Figure 10A**), while those of SlASAT1 remain fairly unaltered in response to the different stress treatments (**Figure 10B**). The observed upregulation of *SlPSAT1* in tomato plants exposed to osmotic stress is consistent with the marked increase of SE levels induced in *Avena sativa* as a result of water deficit stress (Liljenberg and Kates, 1985; Dyas and Goad, 1993), while the downregulation of this gene in response to cold is in accordance with the high SE levels reported in the Arabidopsis chilling-sensitive mutant *chs1* (Hugly et al., 1990). These data, together with the altered response to *Phytophtora infestans* reported in the Arabidopsis *erp1*/*psat1* mutant impaired in SE synthesis (Kopischke et al., 2013) and the induction of *SlPSAT1* observed in tomato plants after MeJ treatment (**Figure 10A**), suggest that SlPSAT1 might play a role in the plant adaptive responses to stress, although the biological significance of its transcriptional response remains to be established. In fact, the widespread expression of *SlPSAT1* and *SlASAT1* points toward a dual role of these sterol acyltrasferases in plant and fruit development, and in plant adaptive responses to stress. Indeed, *SlPSAT1* and *SlASAT1* gene expression is detected in all organs analyzed, including fruits at different developmental and ripening stages (**Figure 6**). Interestingly, *SlPSAT1* and *SlASAT1* show overlapping but largely complementary patterns of expression throughout fruit development. The transcript levels of SlPSAT1 decrease while those of SlASAT1 behave just the opposite way (**Figure 6**), which suggests a concerted transcriptional regulation of these genes in opposite direction throughout fruit development and ripening.

## Author Contributions

AF, TA, and AB conceived, designed, and supervised this study. JL performed all the cloning work and gene expression analysis. JL and AB-M performed mutant phenotype complementation studies. MA, AB-M, and JL carried out GC-MS analysis of sterols. JL, AC, and AB-M conducted the subcellular localization studies. MR and RL synthesized the internal standard and performed chemical structure analysis. JL, AB-M, AC, MA, AF, and TA collected and analyzed data. AF and TA wrote the manuscript.

## Conflict of Interest Statement

The authors declare that the research was conducted in the absence of any commercial or financial relationships that could be construed as a potential conflict of interest. The reviewer DA and handling Editor declared their shared affiliation.

## Footnotes

1 https://imagej.net

2 https://phytozome.jgi.doe.gov/pz/portal.html
