# Supplementary material for: Identification and Characterization of Sterol Acyltransferases Responsible for Steryl Ester Biosynthesis in Tomato
Source: Front Plant Sci. 2018 May 8;9:588. doi: 10.3389/fpls.2018.00588 (PMC5952233; doi:10.3389/fpls.2018.00588)
Supplement: Supplementary file 1 [file Table_1.DOC]

Supplemental Table 1

**Supplemental Table 1:** *SlPSAT1*, *SlASAT1*, *AtASAT1* and *AtPP2AA3* gene specific primers used in this study. The CACC sequence added to the 5’ end of the forward primers to facilitate directional Gateway recombination-based cloning of the amplified sequences is underlined and the ATG start codons are shown in bold.

| **Primer name** | **Primer sequence** | **Application** |
| --- | --- | --- |
| SIPSAT1-fw | CACCGTTTCTAGCCA**ATG**AGAGGA | Amplification of SlPSAT1, SlASAT1 and AtASAT1 open reading frames for cloning into pENTR/D-TOPO vector. |
| SIPSAT1-rev | CCGCCAAACAAAGTTTCTTAAATATCACAC |
| SIASAT1-fw | CACCCAGTTATATT**ATG**GAGGGA |
| SIASAT1-rev | TTATCTCGAAAATGTTGATTTCACAGC |
| AtASAT1-fw | CACCA**ATG**GCGAGTTTCAT |
| AtASAT1-rev | CAGGGTTAAAAAAGATATGCG |
| SlPSAT1-tr-fw | AGCCAATGAGAGGAGGACAC | Expression of SlPSAT1 and SlASAT1 in Arabidopsis *psat1-2* and *asat1-1* T-DNA mutants. |
| SlPSAT1-tr-rev | CAAATGTATTGAACATCAGGCG |
| SlASAT1-tr-fw | GACCAAGGTCCACTTTCTGATTC |
| SlASAT1-tr-rew | GGGCCTAAGGATACGAGTGAC |
| SlPSAT1-qP-fw | TGCTATTGGGATTACGGGAAAG | Determination of SlPSAT1 and SlASAT1 mRNA levels in tomato organs by RT-qPCR. |
| SlPSAT1-qP-rev | GTGTGACAGGATGTGAGATGTAG |
| SlASAT1-qP-fw | GAAGTTAGTTGAAAATGGTTCTAGTG |
| SlASAT1-qP-rev | TTGACCTTTGTTGGACTTTGC |
| C-SlPSAT1-fw | CACC**ATG**AGAGGAGGACACGTGG | Amplification of SlPSAT1, SlASAT1 and AtASAT1 coding sequences to generate C-terminal fusions with GFP/YFP. |
| C-SlPSAT1-rev | CACATAGTGTGACAGGATGT |
| C-SlASAT1-fw | CACC**ATG**GAGGGAAATATTAAAGATG |
| C-SlASAT1-rev | TCTCGAAAATGTTGATTTCACAGC |
| C-AtASAT1-fw | CACC**ATG**GCGAGTTTCAT |
| C-AtASAT1-rev | AAAAAGATATGCGGTCAGTTTCC |
| AtPP2AA3-fw | TAACGTGGCCAAAATGATGC | Amplification of Arabidopsis PP2AA3 reference gene |
| AtPP2AA3-rev | GAAGCCAACATTAACATTAGTAGC |
