# Supplementary material for: Identification and Characterization of Sterol Acyltransferases Responsible for Steryl Ester Biosynthesis in Tomato
Source: Front Plant Sci. 2018 May 8;9:588. doi: 10.3389/fpls.2018.00588 (PMC5952233; doi:10.3389/fpls.2018.00588)
Supplement: Supplementary file 2 [file Table_2.DOC]

Supplemental Table 2

Overall identity percentages shared among de eight tomato ASAT-like candidate proteins and the *Arabidopsis thaliana* ASAT1 (AAQ65159) (Chen et al., 2007). Percentages were calculated from pairwise alignment of the corresponding amino acid sequences using the BLASTP algorithm at the NCBI.

| Gene ID | Solyc11g012260 | Solyc11g012210 | Solyc11g012230 | Solyc11g012250 | Solyc11g012240 | Solyc11g012200 | Solyc11g012220 | Solyc12g089050 |
| --- | --- | --- | --- | --- | --- | --- | --- | --- |
| At3g51970 | 48.9 | 44.6 | 44.6 | 44.2 | 43.4 | 43.1 | 42.1 | 32.9 |
| Solyc11g012260 | -- | 49.0 | 49.0 | 47.5 | 47.0 | 46.7 | 43.4 | 32.6 |
| Solyc11g012210 | -- | -- | 80.4 | 72.2 | 72.2 | 75.3 | 73.4 | 32.2 |
| Solyc11g012230 | -- | -- | -- | 77.9 | 77.6 | 81.9 | 77.9 | 34.2 |
| Solyc11g012250 | -- | -- | -- | -- | 79.4 | 72.2 | 68.8 | 33.0 |
| Solyc11g012240 | -- | -- | -- | -- | -- | 75.3 | 71.1 | 31.2 |
| Solyc11g012200 | -- | -- | -- | -- | -- | -- | 82.4 | 33.9 |
| Solyc11g012220 | -- | -- | -- | -- | -- | -- | -- | 31.6 |
