# Supplementary material for: Identification and Characterization of Sterol Acyltransferases Responsible for Steryl Ester Biosynthesis in Tomato
Source: Front Plant Sci. 2018 May 8;9:588. doi: 10.3389/fpls.2018.00588 (PMC5952233; doi:10.3389/fpls.2018.00588)
Supplement: Supplementary file 3 [file Table_3.PDF]

Supplemental Table 3

Prediction of signal peptide presence in the amino acid sequence of SlPSAT1 using SignalP 4.1 Server (http://www.cbs.dtu.dk/services/SignalP/)

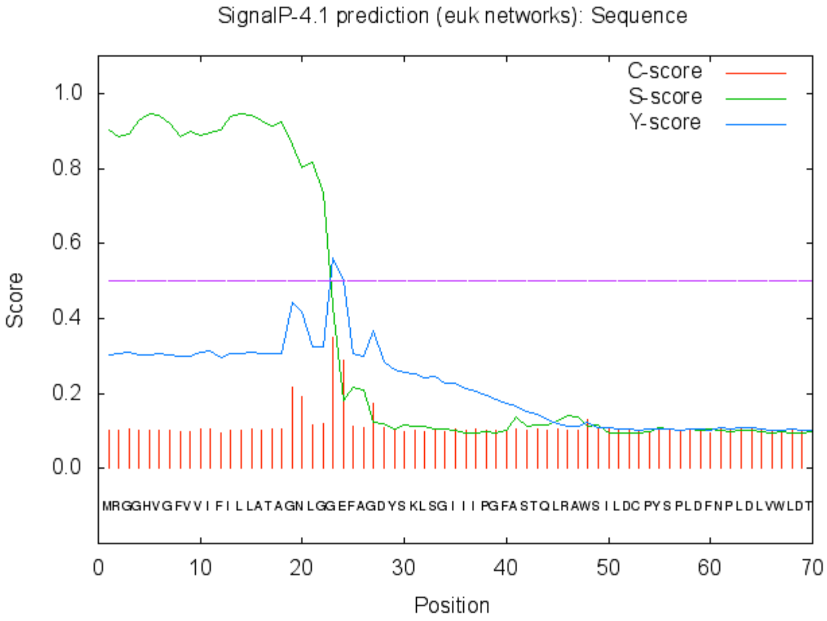

Measure	Position	Value	Cutoff	Signal peptide?
Max. C	23	0.351		
Max. Y	23	0.561		
Max. S	14	0.946		
Mean S	1-22	0.895		
D	1-22	0.742	0.450	YES

Cleavage site between pos. 22 and 23: NLG-GE D=0.742 D-cutoff=0.450 Networks=SignalP-noTM
