# Supplementary material for: Identification and Characterization of Sterol Acyltransferases Responsible for Steryl Ester Biosynthesis in Tomato
Source: Front Plant Sci. 2018 May 8;9:588. doi: 10.3389/fpls.2018.00588 (PMC5952233; doi:10.3389/fpls.2018.00588)
Supplement: Supplementary file 4 [file Table_4.DOC]

Supplemental Table 4

Total free sterol (FS) content (μg/g dry weight) in rosette leaves, seedlings and seeds of Arabidopsis wild type, *asat1-1* and *asat1-1* overexpressing tomato ASAT1. FS content includes cholesterol, brassicasterol, campesterol, stigmasterol, β-sitosterol, isofucosteol, cycloartenol and 24-methylene cycloartanol, and was determined as described in “Material and Methods” section. Values are means ± SD (n=3).

| **Sample** | **wild type** | **asat1-1** | **asat1-1 Pro35S::SlASAT1** |
| --- | --- | --- | --- |
| Leaves | 1.57 ± 0.09 | 1.99 ± 0.06 | 2.05 ± 0.31 |
| Seedlings | 5.17 ± 0,62 | 5.01 ± 0.14 | 5.18 ± 0.54 |
| Seeds | 0.97 ± 0.02 | 0.97 ± 0.06 | 0.92 ± 0.11 |
