# Supplementary material for: Identification and Characterization of Sterol Acyltransferases Responsible for Steryl Ester Biosynthesis in Tomato
Source: Front Plant Sci. 2018 May 8;9:588. doi: 10.3389/fpls.2018.00588 (PMC5952233; doi:10.3389/fpls.2018.00588)
Supplement: Supplementary file 5 [file Table_5.DOC]

Supplemental Table 5

Prediction of subcellular localization of AtASAT1 and SlASAT1 according to version 1.04 of Predotar (<https://urgi.versailles.inra.fr/predotar/>)

| **Protein** | **Mitochondria** | **Plastid** | **Endoplasmic reticulum (ER)** | **Elsewhere** | **Prediction** |
| --- | --- | --- | --- | --- | --- |
| AtASAT1 | 0.03 | 0.00 | 0.99 | 0.01 | ER |
| SlASAT1 | 0.01 | 0.00 | 0.03 | 0.96 | None |
