# Supplementary material for: Identification and Characterization of Sterol Acyltransferases Responsible for Steryl Ester Biosynthesis in Tomato
Source: Front Plant Sci. 2018 May 8;9:588. doi: 10.3389/fpls.2018.00588 (PMC5952233; doi:10.3389/fpls.2018.00588)
Supplement: Supplementary file 6 [file Data_Sheet_1.DOC]

**SUPPLEMENTARY MATERIAL**

**Synthesis of palmitoyl-cholestanol**

Palmitoyl-cholestanol was synthesized according to the procedure described by Yu et al. (2012). Briefly, cholestanol (2.0 g, 5 mmol), palmitic acid (1.39 g, 6 mmol), DCC (0.96 g, 5 mmol), DMAP (56 mg, 0.5 mmol) and dry THF (40 mL) were mixed in a 250 mL round-bottom flask and stirred for 36 h at room temperature. The floating solid was filtrated off and the solvent was concentrated. Then, the concentrated mixture was poured into 500 mL distilled water with fierce stirring. After washing twice, the white sediment was collected and recrystallized from ethanol to yield 2.16 g (74%) of desired product as a white solid, with a purity ≥ 90% (contaminated with ≤ 10% of cholestanol).

The spectroscopy was consistent with the expected structure and matched the published data. Relevant peaks: ^1^H NMR (400 MHz, CDCl_3_) δ 4.70 (td, *J* = 11.3, 5.6 Hz, 1H), 2.25 (t, *J* = 7.5 Hz, 2H), 0.64 (s, 3H).

Screenshot of the ^1^H-NMR (CDCl_3_, 400 MHz):


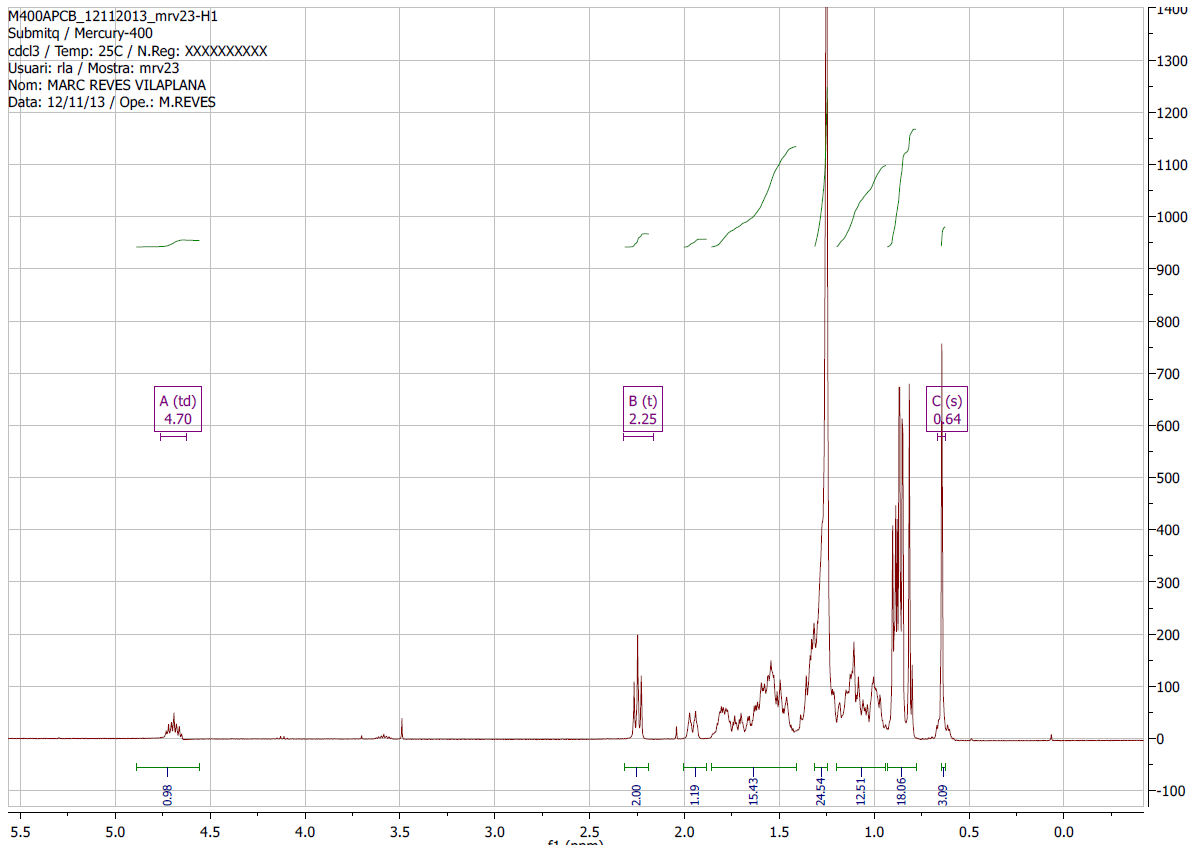


Yu, Y-L., Bai, J-W., and Zhang J-H. (2012). Synthesis and characterization of side-chain cholesterol derivatives based on double bond. *J. Mol. Struct.* 1019, 1-6. doi: 10.1016/j.molstruc.2012.03.034
