# Supplementary figures and images for: Identification and Characterization of Sterol Acyltransferases Responsible for Steryl Ester Biosynthesis in Tomato
Source: Front Plant Sci. 2018 May 8;9:588. doi: 10.3389/fpls.2018.00588 (PMC5952233; doi:10.3389/fpls.2018.00588)

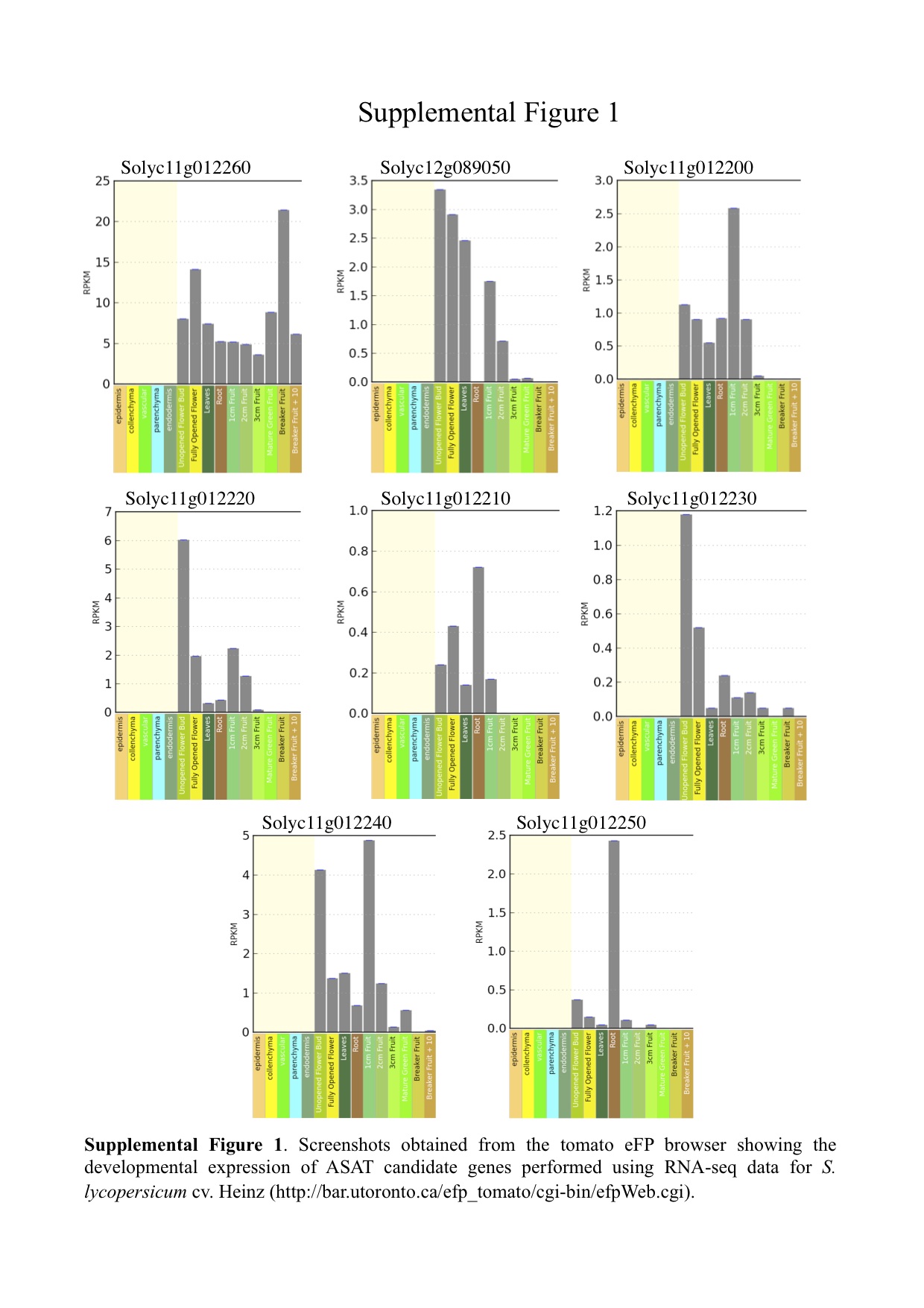

Supplement: Supplementary file 7 [file Image_1.JPEG]

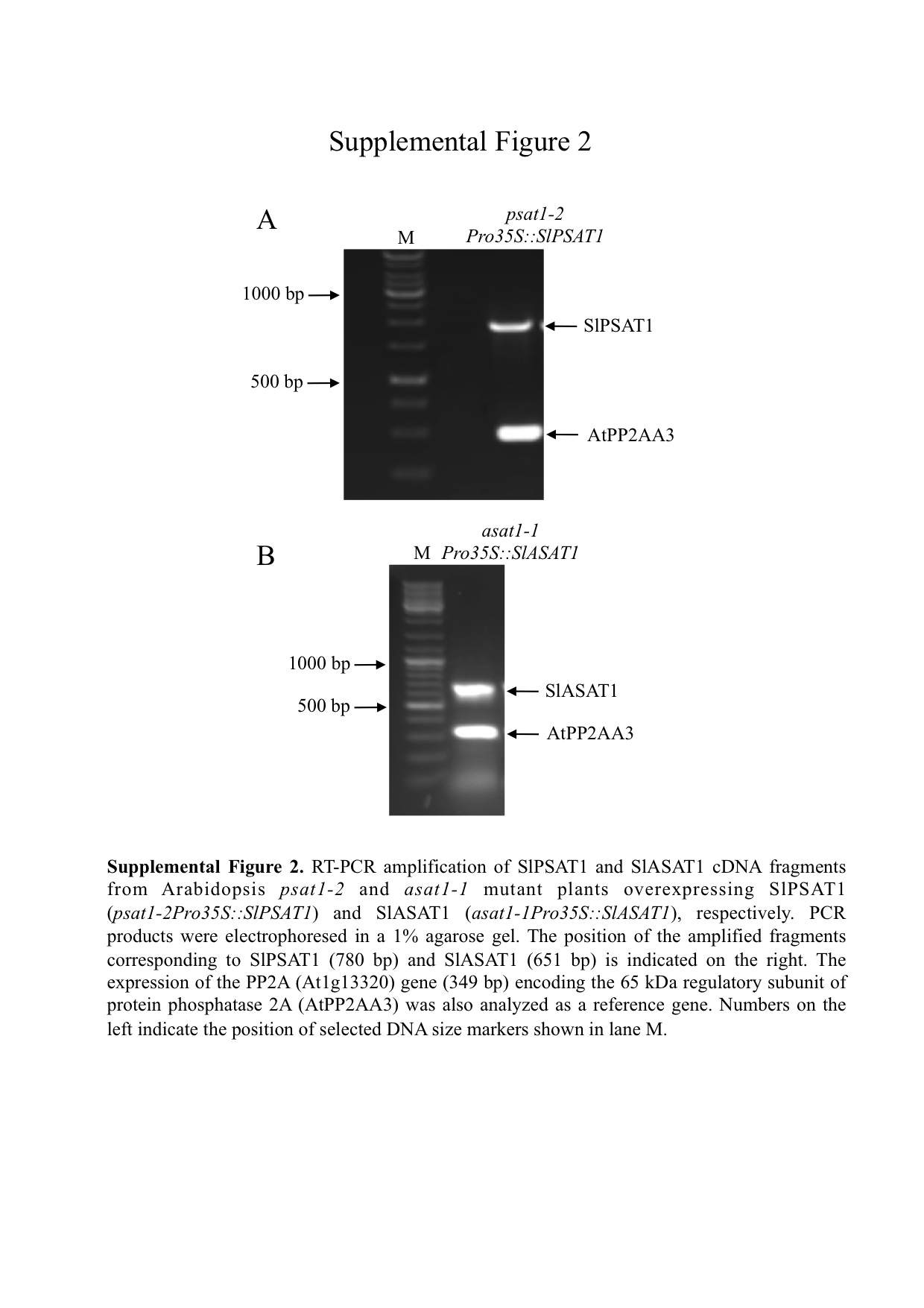

Supplement: Supplementary file 8 [file Image_2.JPEG]

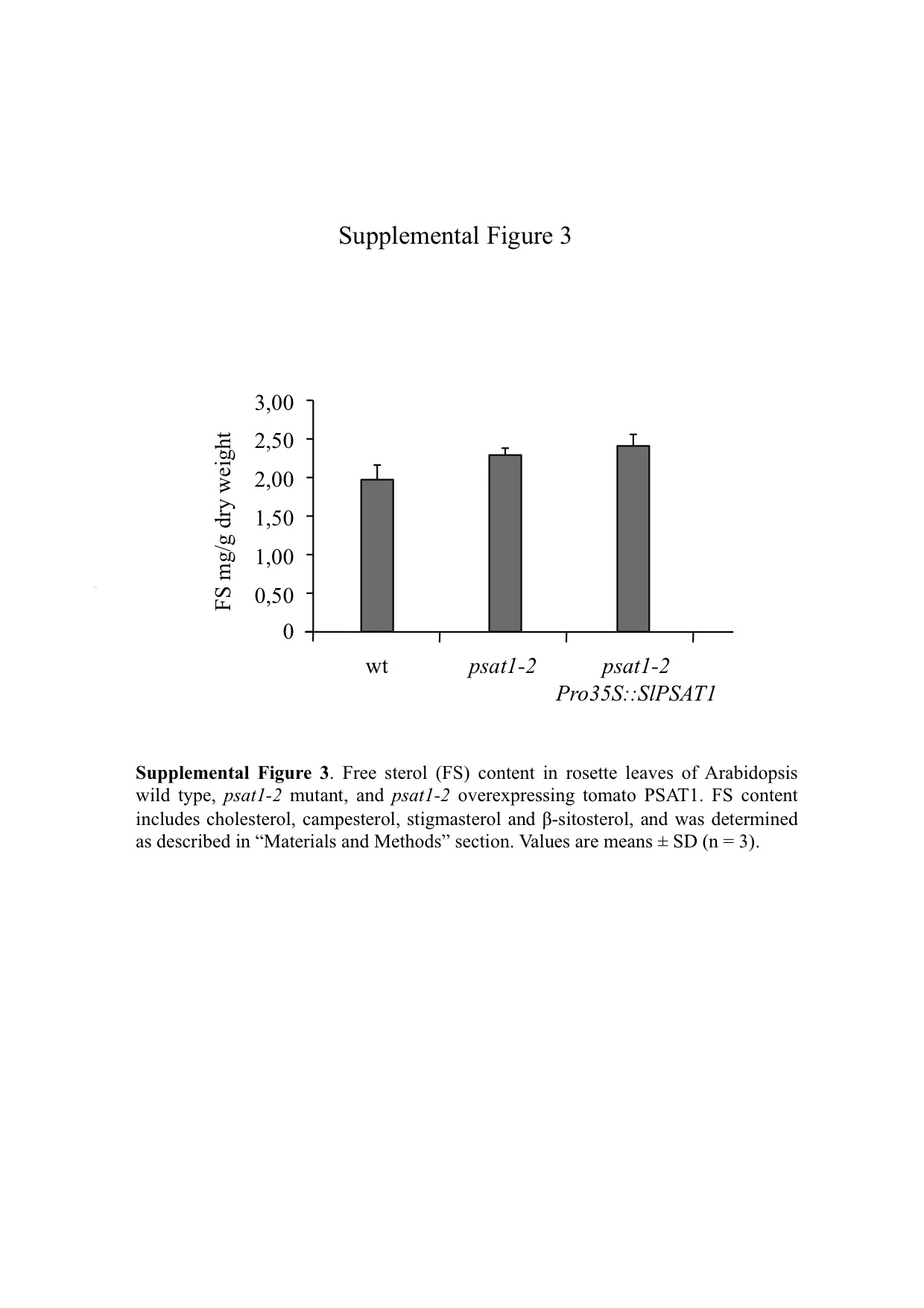

Supplement: Supplementary file 9 [file Image_3.JPEG]

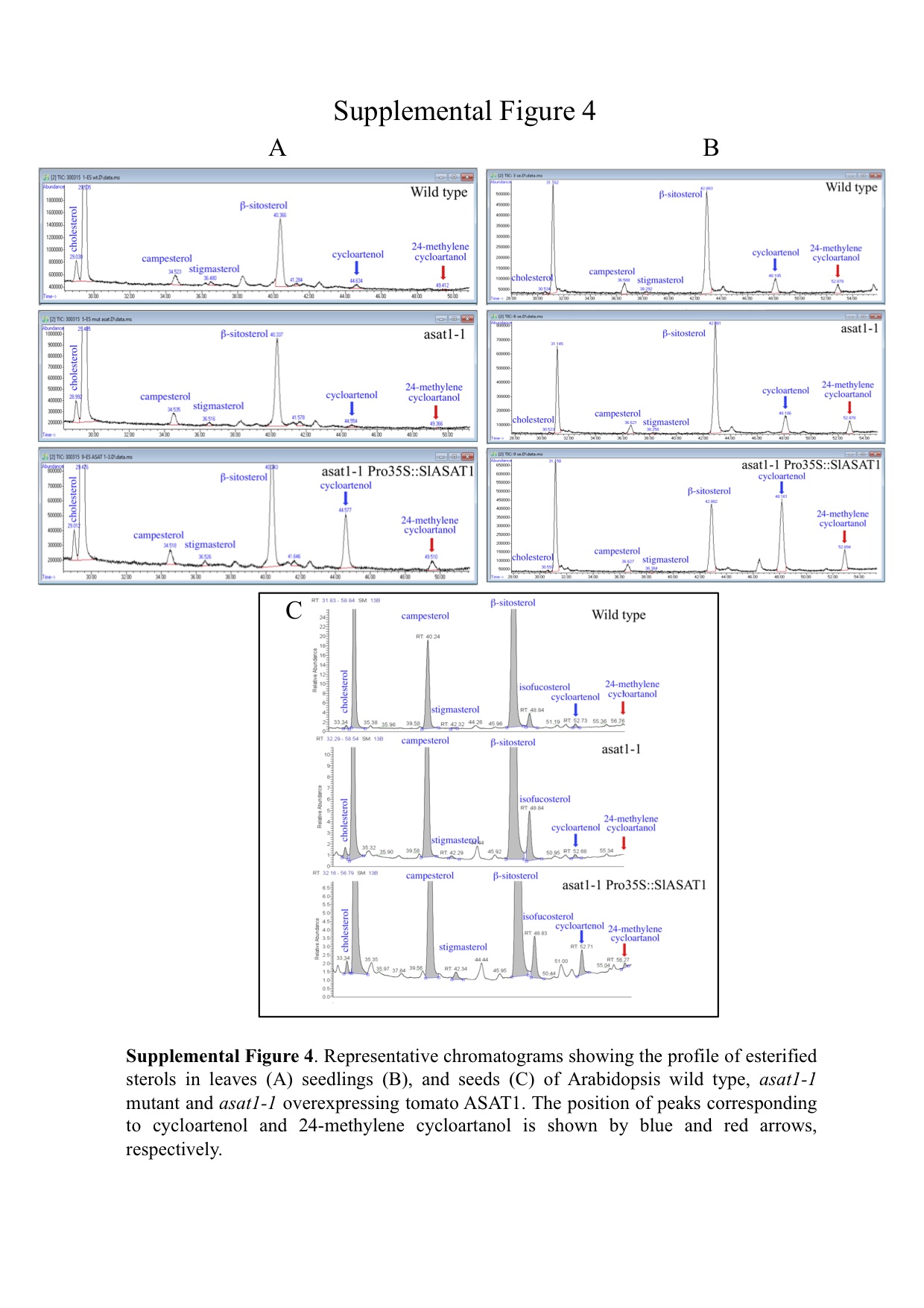

Supplement: Supplementary file 10 [file Image_4.JPEG]

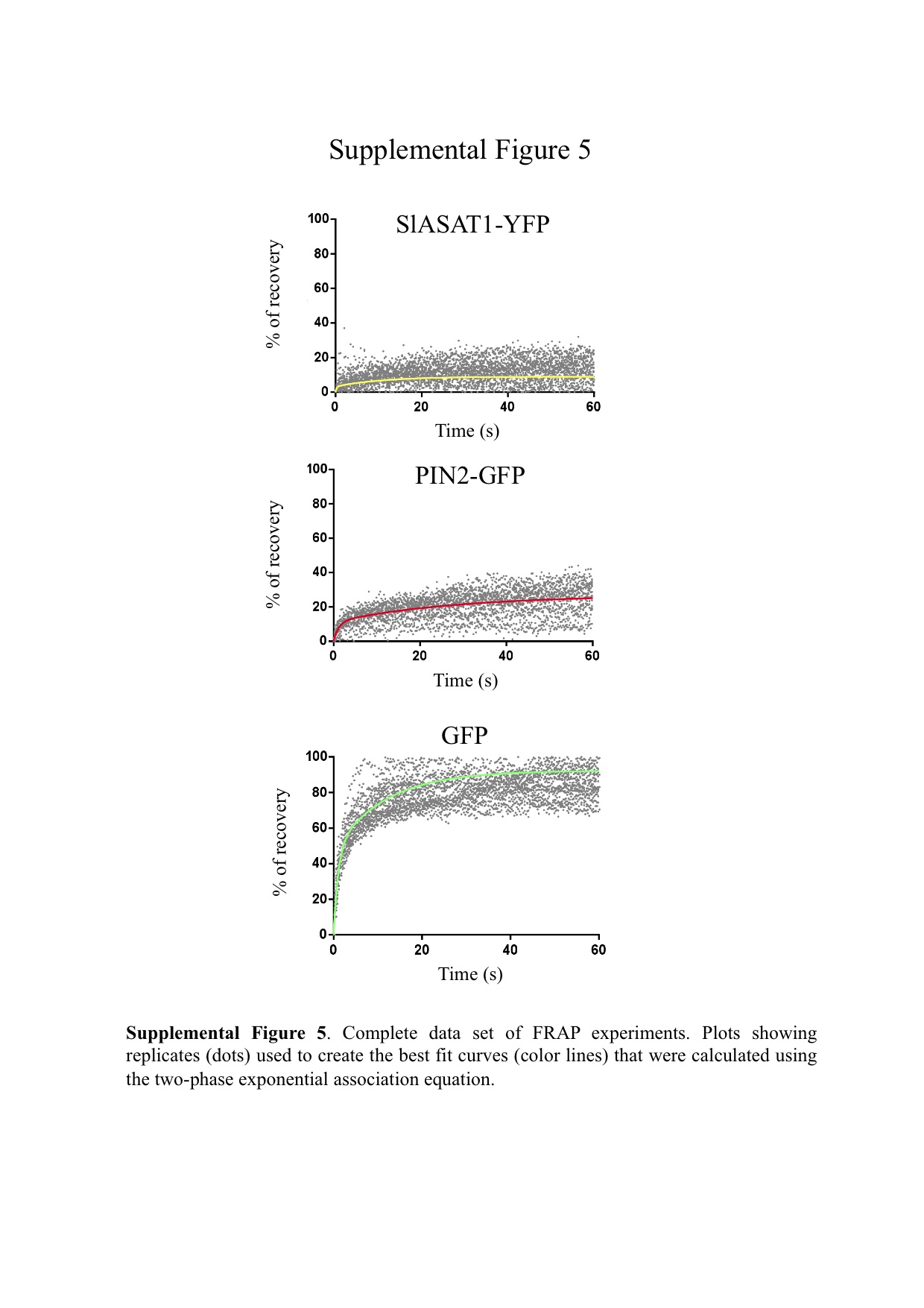

Supplement: Supplementary file 11 [file Image_5.JPEG]
